# Synthesis of Curcumin Analogs Targeting Androgen Receptor for the Selective Inhibition of Castration‐Resistant Prostate Cancer Cells

**DOI:** 10.1111/cbdd.70418

**Published:** 2026-10-15

**Authors:** Manman Huang, Ziyi Chen, Ruiqi Zhang, Shengping Liu, Defeng Xu, Bing Li, Hang Hu

**Affiliations:** ^1^ School of Pharmacy Changzhou University Changzhou P. R. China

**Keywords:** androgen receptor, curcumin analogs, prostate cancer, selectivity, structure–activity relationship

## Abstract

In the present study, a series of curcumin analogs (A1–P3) were designed and synthesized for the inhibition of prostate cancer cells by targeting androgen receptor (AR). All synthesized compounds were structurally characterized by ^1^H NMR, ^13^C NMR, and high‐resolution mass spectrometry (HRMS). In vitro cytotoxicity assays demonstrated that multiple compounds exhibited comparable or improved cytotoxicity against AR‐positive 22Rv1 cells as compared to dimethylcurcumin (DMC, ASC‐J9). Among these compounds, compounds A2, A3, B1–B3, D1, F1, F3, G1, G3, H1, and P3 possessed higher selectivity toward prostate cancer cells as compared to DMC. Compounds O1–O3, A1–A3, B1–B3, F1, F3, G1, I1, and P3 exhibited lower cytotoxicity against AR‐negative PC‐3 cells than those against 22Rv1 cells. Based on the cytotoxicity results, the structure–activity relationships were analyzed. In the AR protein suppression assay, compounds O1–O3, A1–A3, and I1 exhibited similar inhibitory effects on both full‐length AR (AR‐FL) and AR splice variant 7 (AR‐V7) as compared to DMC, while compound P3 exhibited significantly enhanced AR‐FL suppression effect as compared to DMC. In comparison, compounds B2, B3, F1, and F3 exhibited reduced AR‐FL and AR‐V7 suppression effects as compared to DMC; B1 and G1 exhibited no significant suppression effect on AR‐FL and AR‐V7, suggesting that these compounds might inhibit prostate cancer cells by multiple mechanisms. Molecular docking simulations further confirmed that compounds O1–O3, A1–A3, I1, and P3 displayed high targeting ability to AR. The probable binding modes of these compounds to AR were also revealed. By modifying the molecular structure of curcumin, this study identified several compounds (A2, A3, P3) with high inhibitory activity and selectivity against castration‐resistant prostate cancer cells by targeting AR‐FL and AR‐V7. These three compounds are promising new candidates for castration‐resistant prostate cancer drug discovery.

## Graphical abstract

This study identified several compounds (A2, A3, P3) with high inhibitory activity and selectivity against prostate cancer cells by targeting AR‐FL and AR‐V7. These three compounds are promising new candidates for prostate cancer drug discovery.

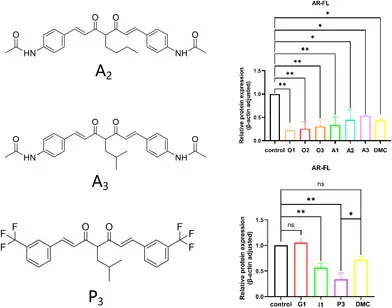

## 1 Introduction

Curcumin is one of the most representative active constituents in the curcumin family, mainly isolated from the rhizomes of *Curcuma longa* L., and is currently one of the most extensively studied natural polyphenolic compounds (Esmaeli et al. 2025). Previous studies have confirmed that curcumin possesses various pharmacological activities, including antitumor, anti‐inflammatory (Peng et al. 2021; Termini et al. 2020; Wang et al. 2023), antioxidant, antibacterial, and antiviral effects (Jamil et al. 2023; Moghadamtousi et al. 2014). It can inhibit the proliferation, invasion, and metastasis of tumor cells by regulating multiple signaling pathways such as NF‐κB, STAT3, and WNT/β‐catenin (Abadi et al. 2022; Joshi et al. 2023; Lin et al. 2018), and induce tumor cell apoptosis through the mitochondrial pathway (Shi et al. 2009), showing great potential in the prevention and treatment of malignant tumors (Gutsche et al. 2025). However, natural curcumin suffers from poor water solubility (Howaili et al. 2020), rapid in vivo metabolism, and low oral absorption efficiency, resulting in unsatisfactory bioavailability (Hu et al. 2020; Nejabat et al. 2025). Moreover, its inherent antitumor activity is limited, which greatly restricts its further clinical application (Feltrin et al. 2022). At present, rational modification based on curcumin as a lead structure to design and synthesize novel derivatives with improved activity and druggability has become an important direction in the research and development of novel antitumor agents (Kuzminska et al. 2024; Liu et al. 2026). Therefore, structural optimization of curcumin to design and synthesize novel derivatives with enhanced activity has emerged as a key focus in current drug discovery.

At present, numerous studies on the structural modification of curcumin have been conducted in the academic community. Dimethylcurcumin (DMC) can selectively promote the degradation of the androgen receptor (AR) (Cheng et al. 2018), effectively inhibit the activation of the AR signaling pathway (Hu et al. 2021), and exhibit significant anti‐proliferative effects on castration‐resistant prostate cancer cells (Lai et al. 2021). Studies have shown that DMC can not only degrade full‐length androgen receptors but also target AR splice variants and other drug‐resistant mutants (Hu and Xu 2022; Tomeh et al. 2019), while inhibiting STAT3 phosphorylation through non‐AR‐dependent pathways. Liu et al. reported a series of curcumin derivatives as AR inhibitors, among which several compounds showed excellent inhibitory effects on 22Rv1 cells (Liu et al. 2024). Zhou et al. (2016) reported 12 pyridine analogs of curcumin, among which the FN group exhibited the strongest inhibitory effects on the growth of 22Rv1 cells and androgen receptor activation, and could serve as candidate anti‐prostate cancer agents targeting the androgen receptor signaling pathway. Zhou et al. (2014) reported five curcumin analogs with different linkers that could inhibit the growth of 22Rv1 and LNCaP cells, induce apoptosis, and block androgen receptor activation. Gong et al. (2024) reported 20 curcumin derivatives containing halogen or nitrogen atoms, proving that multiple compounds exhibited better inhibitory activity against 22Rv1 cells than dimethylcurcumin. Belluti et al. (2019) reported novel curcumin derivatives with phthalimide moieties, evaluated their improved stability, pharmacodynamics, and free radical scavenging capacity, and determined the anti‐proliferative activity of the synthesized molecules against aggressive prostate tumor cells. Based on the above studies, curcumin derivatives show promising application prospects in inhibiting the progression and metastasis of castration‐resistant prostate cancer (Hu et al. 2019; Wang et al. 2016; Yamashita et al. 2012).

To further obtain novel curcumin derivatives with superior activity and improved druggability against prostate cancer, curcumin was used as the lead compound, and structural modifications were performed on its benzene‐ring substituents and β‐diketone moiety in the present study. A series of curcumin analogs were designed and synthesized. In vitro cytotoxicity assays were employed to evaluate the inhibitory effects of the target compounds on both human prostate cancer cell line and human sebaceous gland cell line. Based on the cytotoxicity results, the structure–activity relationships were analyzed. Compounds with favorable cytotoxicity against prostate cancer cells were further studied regarding the suppression effects on the expression of AR protein in prostate cancer cells. At last, molecular docking simulations were conducted in order to investigate their binding affinity and binding modes with the AR protein.

## 2 Materials and Methods

### 2.1 Materials

2,4‐Pentanedione (acetylacetone), bromomethylcyclopropane, n‐butyl bromide, isobutyl bromide, boric acid, n‐butylamine, tributyl borate, and substituted benzaldehydes are all commercially available reagents and were used directly without further purification. All solvents used were of analytical grade.

### 2.2 Synthesis of Curcumin Analogs

The reaction is carried out in two steps. In the first step, acetylacetone (5 g, 49.94 mmol), bromomethylcyclopropane (8.09 g, 59.93 mmol), and potassium carbonate (13.8 g, 99.98 mmol) were dissolved in 50 mL of DMF, and the mixture was stirred at 60°C for 6 h. After the reaction was complete, the solvent was evaporated under reduced pressure, the residues were dissolved in distilled water, and extracted twice with ethyl acetate. The combined organic phases were washed with saturated NaCl solution and dried over anhydrous magnesium sulfate, then filtered. The filtrate was concentrated and 3‐(cyclopropylmethyl)pentane‐2,4‐dione was obtained by vacuum distillation as a pale‐yellow transparent liquid (5.5 g, yield 71.4%). By replacing bromomethylcyclopropane with 1‐bromobutane, 3‐butylpentane‐2,4‐dione (5.2 g, yield 66.6%) was synthesized using the same method. By replacing bromomethylcyclopropane with 2‐bromoisobutane, 3‐isobutylpentane‐2,4‐dione (6.1 g, yield 78.1%) was also synthesized using the same method.

In the second step, substituted acetylacetone (3.2 mmol) and boric acid (0.098 g, 1.6 mmol) were dissolved in N, N‐dimethylformamide (5 mL). The solution was then heated to 80°C for 15 min. Subsequently, tributyl borate (1.47 g, 6.4 mmol) and substituted benzaldehyde (6.4 mmol) were added, followed by stirring for about 15 min. Then n‐butylamine (0.117 g, 1.6 mmol) was added dropwise. The reaction was continued at 80°C for 2 h. After that, 20% acetic acid (30 mL) was added and stirred for 1 h. After cooling to room temperature, the solid was filtered and recrystallized from methanol to obtain the yellow powdery target product. The synthesized compounds were characterized by ^1^H NMR, ^13^C NMR spectra, and HRMS. The purities of all synthesized compounds were determined by high performance liquid chromatography, since trace impurities would potentially interfere with cellular activity results.

### 2.3 In Vitro Cytotoxicity Tests

22Rv1 cells are AR‐positive prostate cancer cells, which express androgen receptor and are suitable for evaluating the activity of AR‐targeted inhibitors. In contrast, PC‐3 cells are AR‐negative prostate cancer cells lacking functional androgen receptor expression. A comparison of compound cytotoxicity between these two cell lines can help preliminarily verify whether the antiproliferative effect of tested compounds is associated with AR signaling. 22Rv1 and PC‐3 human prostate cancer cells were cultured in RPMI 1640 medium. SZ95 human sebaceous gland cells were cultured in DMEM medium. Both media were supplemented with 10% fetal bovine serum, 100 units/mL penicillin, and 100 μg/mL streptomycin. Cells were cultured at 37°C in a 5% carbon dioxide atmosphere. The MTT assay was used to determine the in vitro cytotoxicity of the synthesized compounds. Briefly, 22Rv1, PC‐3 cells, and SZ95 cells were seeded in 96‐well plates at a density of 5 × 10^3^ cells per well, and cultured at 37°C in a 5% carbon dioxide atmosphere. The synthesized compounds and DMC were applied to the cells at concentrations of 1.25, 2.5, 5, 10, 20, and 40 μM for 24 h. Then, cell viability was measured by standard MTT assays. The half‐maximal inhibitory concentration (IC_50_) of each compound on 22Rv1, PC‐3, and SZ95 cells was calculated based on the cell viability data.

### 2.4 Molecular Docking Simulations

In this study, molecular design and docking experiments were performed using Discovery Studio software. With reference to the molecular structure of curcumin, the target binding protein (PDB ID: 3ZQT) was selected from the Protein Data Bank (PDB). For small‐molecule ligand preparation, all compound structures were first drawn using Chem Draw and imported into the software. Their three‐dimensional geometries were optimized using the “Clean Geometry” function, followed by uniform preprocessing including hydrogenation and energy minimization. The optimized files were saved upon completion. For macromolecular protein preparation, the crystal structure of 3ZQT was imported, water molecules were removed, and protein preprocessing was carried out before saving. In the CDOCKER precision docking stage, the preprocessed protein was imported, the receptor was defined, and the active site was determined. Docking was performed using the CDOCKER algorithm, and calculations were initiated after setting the relevant parameters.

### 2.5 Western Blot

Protein was extracted from 22Rv1 cells, and the protein concentration was determined and normalized using the BCA assay. A 12% separating gel and 5% stacking gel were prepared. Ten microliters of protein sample were loaded per well. Electrophoresis was performed at a constant voltage of 80 V for 30 min, followed by 120 V until the bromophenol blue marker reached the bottom of the separating gel. Proteins were transferred to a PVDF membrane using the wet transfer method at a constant voltage of 100 V for 90 min. The membrane was blocked with 5% skimmed milk at room temperature, then incubated with primary antibody (diluted 1:5000) overnight at 4°C. After washing three times with TBST for 10 min, the membrane was incubated with the corresponding secondary antibody (diluted 1:5000) at room temperature for 1 h, followed by another three washes with TBST. Development was performed using an ECL chemiluminescence kit. The grayscale values of target protein bands were quantitatively analyzed using ImageJ software. β‐actin was used as an internal reference to calculate the relative expression level of the target protein.

### 2.6 Statistical Analysis

All data were presented as the mean ± standard deviations (SD). Statistical significance of differences between groups was evaluated by one‐way analysis of variance (ANOVA) using GraphPad software. Significance was defined as *p* < 0.05.

## 3 Results and Discussion

Starting from acetylacetone, the central alkyl group R_1_ was introduced via alkylation, followed by the introduction of aryl groups R_2_ at both termini through boron‐catalyzed double aldol condensation. The synthetic route of curcumin analogs is illustrated in Scheme 1. Substituted acetylacetone reacts with boron oxide to form a boron complex, which then undergoes condensation with the aldehyde upon addition of substituted benzaldehyde and n‐butylamine. Finally, the boron complex is cleaved by heating in dilute acetic acid to afford the final product. The ^1^H NMR, ^13^C NMR, and HRMS spectra of the synthesized compounds were characterized. The NMR and HRMS data are summarized in part 1 of Supporting Information. The NMR and HRMS spectra are shown in part 2 and part 3 of Supporting Information, respectively. The results confirmed that all compounds were successfully prepared. The chemical structures of the synthesized compounds are shown in Scheme 2.

**SCHEME 1:**
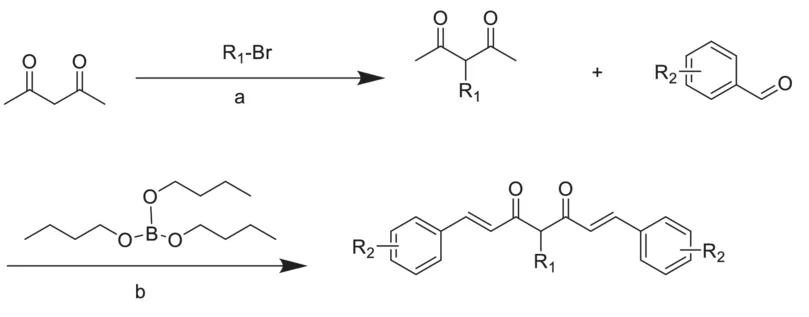
Synthetic scheme of curcumin analogs. R_1_ = cyclopropyl, n‐butyl, isobutyl. R_2_ = fluoro, methoxy, methylsulfonyl, acetylamino, trifluoromethyl. a: K_2_CO_3_; DMF; 60°C; b: H_3_BO_3_; n‐butylamine; DMF; 80°C.

**SCHEME 2:**
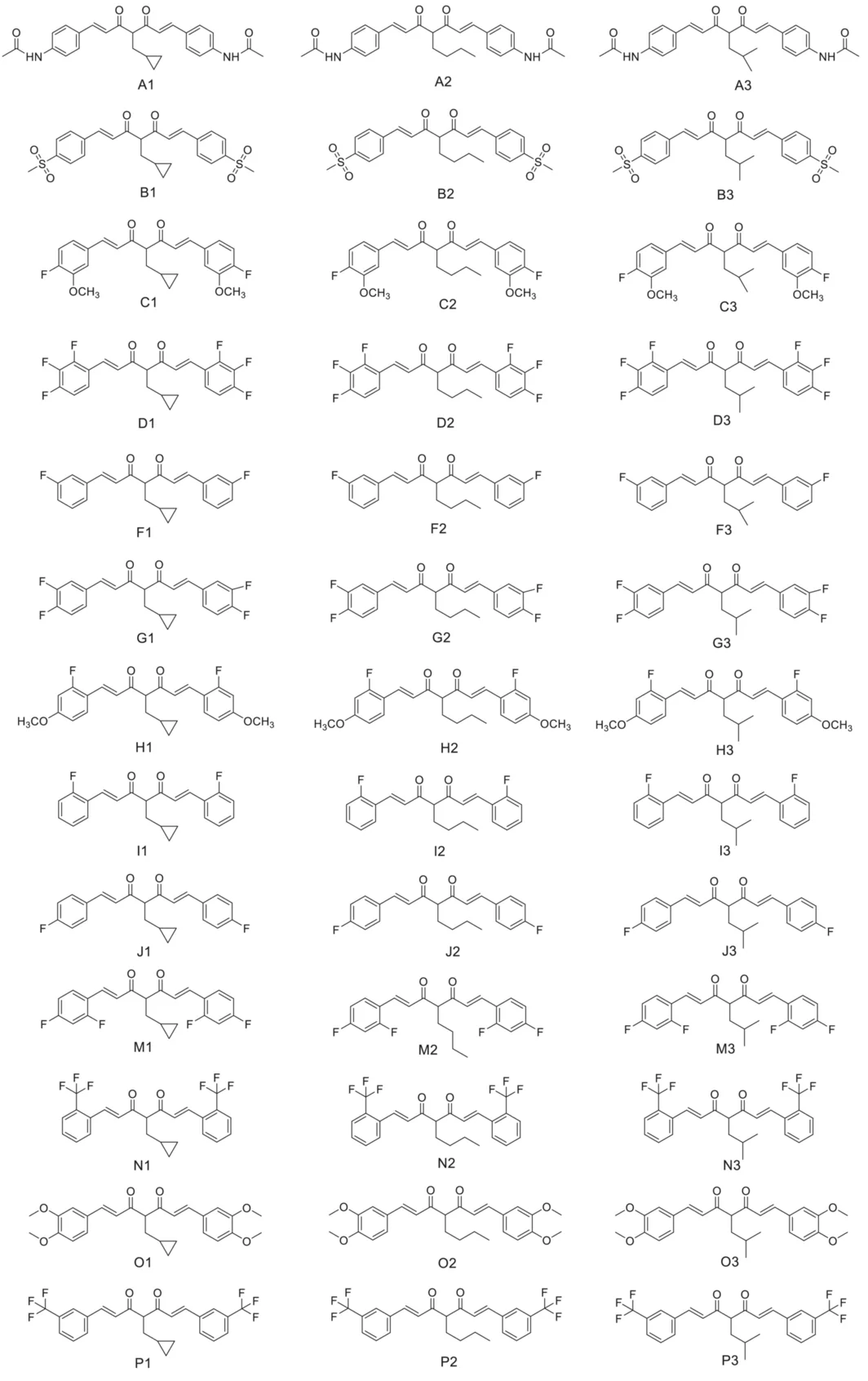
Chemical structure of the synthesized compounds.

The in vitro cytotoxicity of compounds A1–P3 against 22Rv1 human prostate cancer cells was evaluated using the MTT assay. The IC_50_ values were calculated and presented in Table 1. Compounds A1–A3, B1–B3, D1, F1, F3, G1–G3, H1, I1, M1, and P3 exhibited significantly improved cytotoxicity against 22Rv1 cells as compared to DMC, while compounds C1, C3, F2, H2, H3, I2, J1, J3, M3, O1, O2, and O3 displayed slightly enhanced or similar cytotoxicity against 22Rv1 cells as compared to DMC. Other synthesized compounds showed reduced cytotoxicity against 22Rv1 cells. The cytotoxicity of some compounds with good inhibitory effects on 22Rv1 cells was further tested on SZ95 human sebaceous gland cells. As shown in Table 2, compounds A2, A3, B1–B3, D1, F1, F3, G1, G3, H1, and P3 possessed higher selectivity toward prostate cancer cells as compared to DMC. The cytotoxicity of some compounds with good inhibitory effects on 22Rv1 cells was also tested on AR‐negative PC‐3 cells. As shown in Table 3, all tested compounds exhibited lower cytotoxicity against PC‐3 cells than those against 22Rv1 cells, suggesting that these compounds exert their potent cytotoxicity against 22Rv1 cells via targeting AR.

**TABLE 1:** IC_50_ of each compound against 22Rv1 cells after treatment for 24 h.

Compound no.	IC_50_ (μM)
A1	6.88 ± 0.65
A2	1.09 ± 1.38
A3	3.20 ± 1.53
B1	5.93 ± 0.84
B2	1.52 ± 0.96
B3	2.95 ± 1.21
C1	15.76 ± 0.84
C2	25.34 ± 0.96
C3	14.38 ± 1.21
D1	7.36 ± 1.57
D2	30.93 ± 0.86
D3	36.17 ± 0.75
F1	4.11 ± 0.57
F2	13.69 ± 0.73
F3	7.53 ± 0.86
G1	4.64 ± 1.95
G2	8.47 ± 0.62
G3	3.55 ± 1.69
H1	5.77 ± 0.81
H2	13.52 ± 0.79
H3	12.52 ± 1.53
I1	6.81 ± 0.78
I2	12.87 ± 0.58
I3	29.64 ± 1.49
J1	14.25 ± 0.91
J2	22.98 ± 0.58
J3	11.78 ± 0.73
M1	9.74 ± 0.39
M2	33.10 ± 0.38
M3	15.23 ± 1.54
N1	17.37 ± 0.67
N2	45.94 ± 0.47
N3	65.12 ± 0.75
O1	11.29 ± 0.52
O2	14.78 ± 0.91
O3	10.59 ± 0.78
P1	28.37 ± 0.39
P2	32.5 ± 0.65
P3	7.69 ± 0.89
DMC	15.48 ± 0.81

**TABLE 2:** IC_50_ of each compound against SZ95 cells after treatment for 24 h.

Compound no.	IC_50_ (μM)	
	SZ95	SZ95/22Rv1
O1	1.959 ± 0.26	0.174
O2	4.929 ± 0.86	0.333
O3	8.629 ± 0.47	0.815
D1	14.37 ± 0.35	1.953
A1	3.284 ± 1.51	0.477
A2	8.952 ± 0.36	8.213
A3	4.751 ± 0.83	1.487
B1	9.515 ± 0.64	1.604
B2	9.948 ± 0.35	6.558
B3	7.571 ± 0.64	2.569
F1	7.473 ± 0.87	1.818
F3	10.27 ± 0.62	1.365
G1	16.26 ± 0.79	3.507
G2	9.468 ± 0.64	1.118
G3	12.11 ± 0.47	3.413
H1	8.11 ± 0.82	1.405
I1	4.593 ± 0.58	0.675
M1	10.34 ± 0.59	1.062
P3	11.81 ± 0.63	1.537
DMC	17.95 ± 0.82	1.159

**TABLE 3:** IC_50_ of each compound against PC‐3 cells after treatment for 24 h.

Compound no.	IC_50_ (μM)
O1	25.68 ± 0.58
O2	30.69 ± 1.03
O3	23.76 ± 0.47
A1	7.62 ± 1.52
A2	10.27 ± 0.61
A3	18.86 ± 0.56
B1	28.78 ± 0.37
B2	42.56 ± 0.75
B3	46.04 ± 0.56
F1	32.72 ± 0.38
F3	29.97 ± 0.57
G1	31.90 ± 0.98
I1	29.18 ± 1.21
P3	41.86 ± 0.76
DMC	38.27 ± 0.49

The primary structure–activity relationships of the synthesized compounds were elucidated based on their cytotoxicity against 22Rv1 cells. The substituent R_1_ on the β‐diketone moiety has significant influence on the activity. Varying the steric bulk and lipophilicity of R₁ leads to divergent IC₅₀ values, yet no clear universal trend could be established to favor a specific type of R₁ modification. It is speculated that the R₁ group acts as a moderate steric modulator within the AR ligand‐binding pocket. The substituents R_2_ on the benzene ring have drastic influence on the activity of the synthesized compounds. The activity of R_2_ could be roughly ranked as follows: 4‐acetamido, 4‐methanesulfonyl > 3‐fluoro, 3,4‐difluoro > 2‐fluoro‐4‐methoxy, 3,4‐dimethoxy > 4‐fluoro‐3‐methoxy, 2,3,4‐trifluoro, 2‐fluoro, 4‐fluoro, 2,4‐difluoro, 2‐trifluoromethyl, 3‐trifluoromethyl. Notably, phenyl rings bearing electron‐withdrawing groups at the para‐position (4‐acetamido, 4‐methanesulfonyl) afford the most potent analogs, implying favorable electronic interactions between the aromatic scaffold and the AR binding cavity. Multiple fluorine substitutions generally retain moderate activity, while ortho‐trifluoromethyl substituents yield weaker inhibitory effects, which is likely attributed to increased steric hindrance disrupting optimal π‐stacking contacts within the ligand pocket. Methoxy‐substituted derivatives display intermediate activity, indicating that electron‐donating alkoxy groups alone cannot achieve activity comparable to strong para‐electron‐withdrawing substituents. Collectively, both electronic effects and steric properties of terminal R_2_ substituents jointly govern the binding affinity and cellular activity of these compounds.

The inhibitory effects of compounds O1–O3, A1–A3, B1–B3, F1, F3, G1, I1, and P3 on the expression of AR protein were further evaluated and compared with DMC. As shown in Figures 1, 2, 3, O1–O3, A1–A3, F3, I1, and P3 significantly suppressed the AR‐FL and the drug resistance‐related protein AR‐V7 expression levels in 22Rv1 cells; B2, B3, and F1 suppressed AR‐FL but not AR‐V7 significantly, whereas B1 and G1 exhibited almost no AR suppression effects. Among the tested compounds, O1–O3, A1–A3, and I1 exhibited similar AR‐FL and AR‐V7 suppression effects as DMC, while B2, B3, F1, and F3 showed reduced AR‐FL and AR‐V7 suppression effects as compared to DMC, and P3 showed significantly enhanced AR‐FL suppression effect and similar AR‐V7 suppression effect as compared to DMC.

**FIGURE 1:**
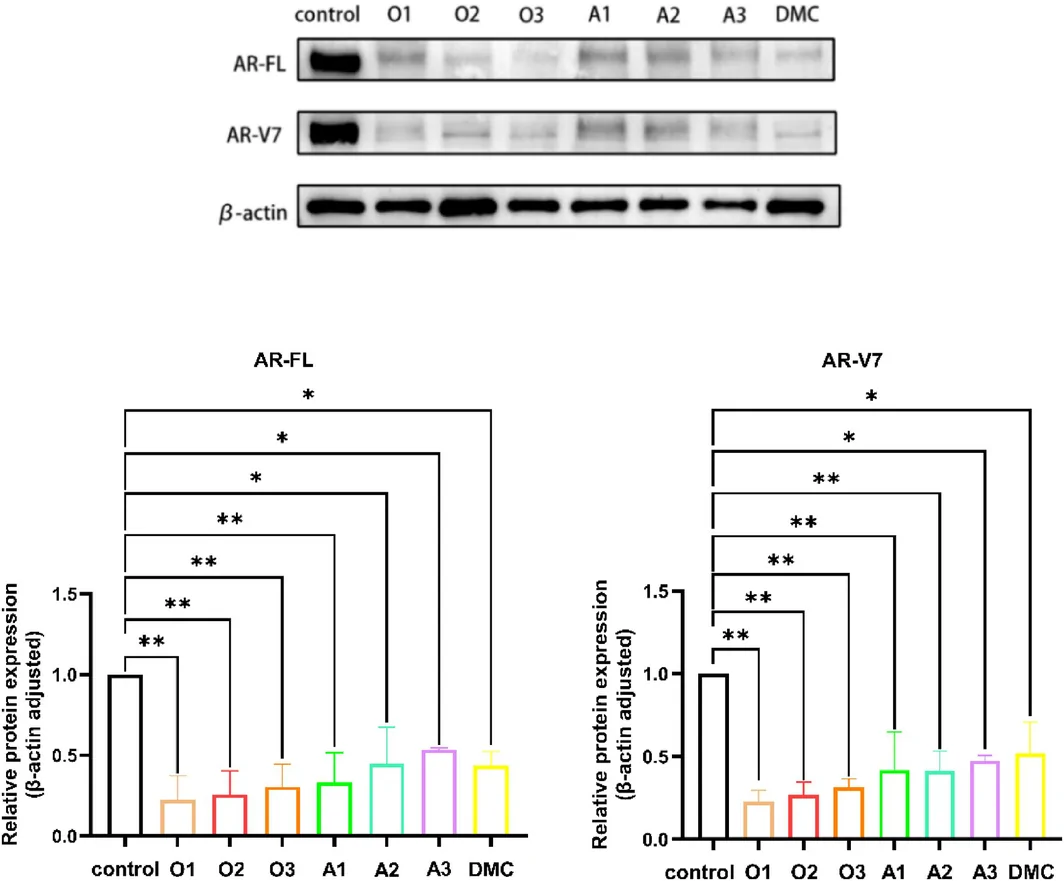
Western blot analysis of suppression effects of O1–O3 and A1–A3 on AR protein expression of 22Rv1 cells. Cells were treated with 5 μM compounds for 24 h. Data represent mean ± SD (*n* = 3).

**FIGURE 2:**
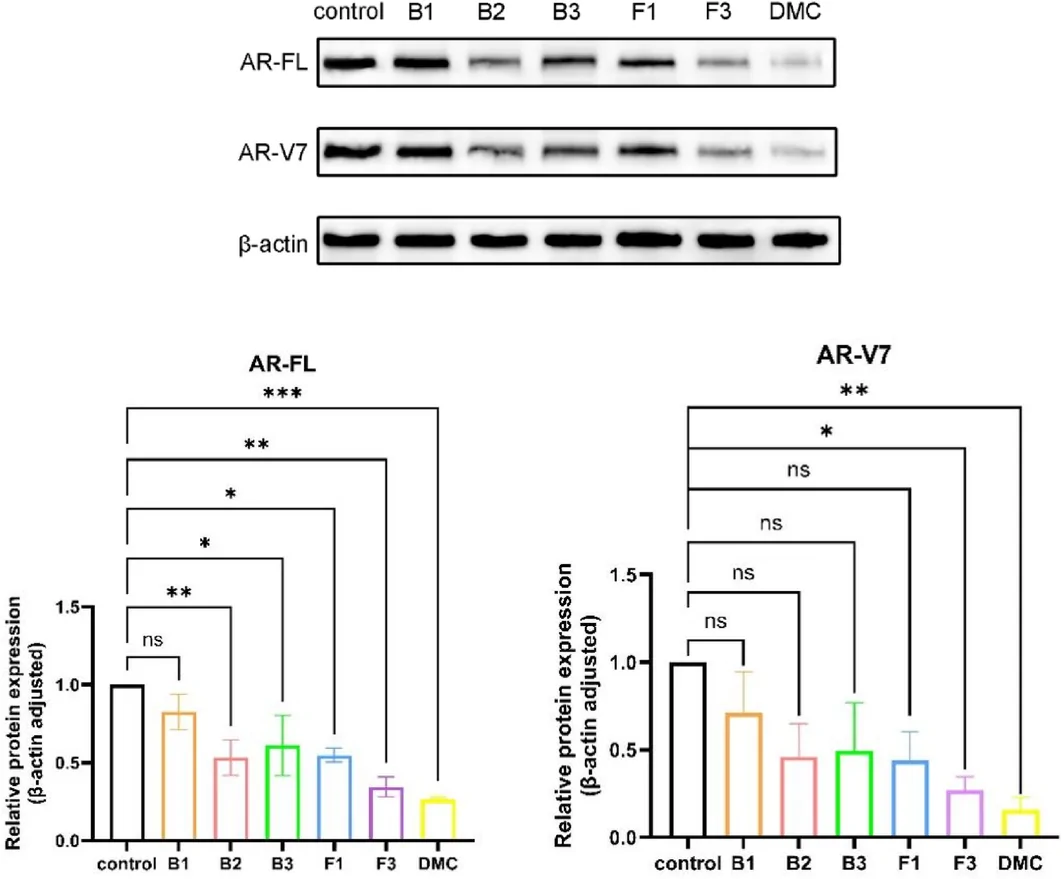
Western blot analysis of suppression effects of B1–B3, F1, and F3 on AR protein expression of 22Rv1 cells. Cells were treated with 5 μM compounds for 24 h. Data represent mean ± SD (*n* = 3).

**FIGURE 3:**
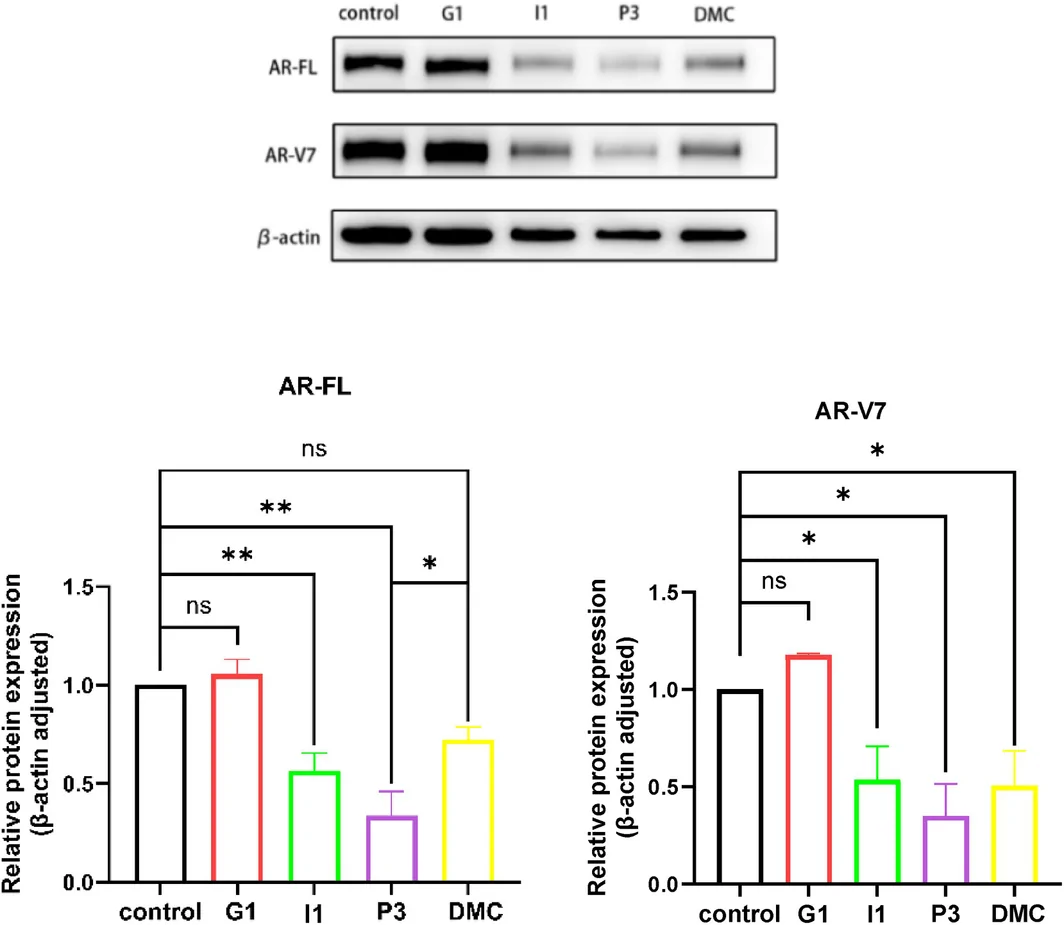
Western blot analysis of suppression effects of G1, I1, P3, and DMC on AR protein expression of 22Rv1 cells. Cells were treated with 5 μM compounds for 24 h. Data represent mean ± SD (*n* = 3).

To investigate the binding affinity and potential binding modes of the tested compounds with AR protein, molecular docking simulations were conducted. The AR crystal structure (PDB code: 3ZQT) was selected for docking analysis. 3ZQT is a complex of the androgen receptor bound with Nordihydroguaiaretic acid (NDGA). NDGA is a natural polyphenolic AR inhibitor. It possesses a highly similar molecular scaffold to the curcumin analogs synthesized in our work, offering a probable binding pocket for our synthesized compounds. Meanwhile, we redocked the native ligand NDGA back into the binding cavity of the AR protein. The calculated root‐mean‐square deviation (RMSD) is 1.62 Å (< 2.0 Å), which confirms that the molecular docking method adopted in this study is viable and the simulation results are credible. DMC and the synthesized compounds were docked into 3ZQT to form new complexes. The ‐CODOCKER interaction energies of different compounds binding to AR are shown in Figure 4. The results showed that compounds A1–A3, B1–B3, O1–O3, G1, and I1 displayed higher binding affinity to AR protein than DMC; compound P3 showed similar binding affinity to AR protein as compared to DMC, whereas compounds F1 and F3 exhibited reduced binding affinity to AR protein. Combined with findings from the AR suppression assay, these results confirmed that A1–A3, O1–O3, I1, and P3 exhibited strong targeting activity toward AR. Compounds B1–B3 exhibited higher binding affinity toward the AR protein than DMC, yet they display reduced AR degradation activity. Robust binding of compounds to the androgen receptor ligand‐binding domain is a necessary but not sufficient prerequisite for initiating AR degradation. Although these derivatives may be capable of occupying the ligand‐binding pocket and forming stable binding complexes, they fail to elicit the critical conformational rearrangements indispensable for the subsequent ubiquitination and degradation cascades.

**FIGURE 4:**
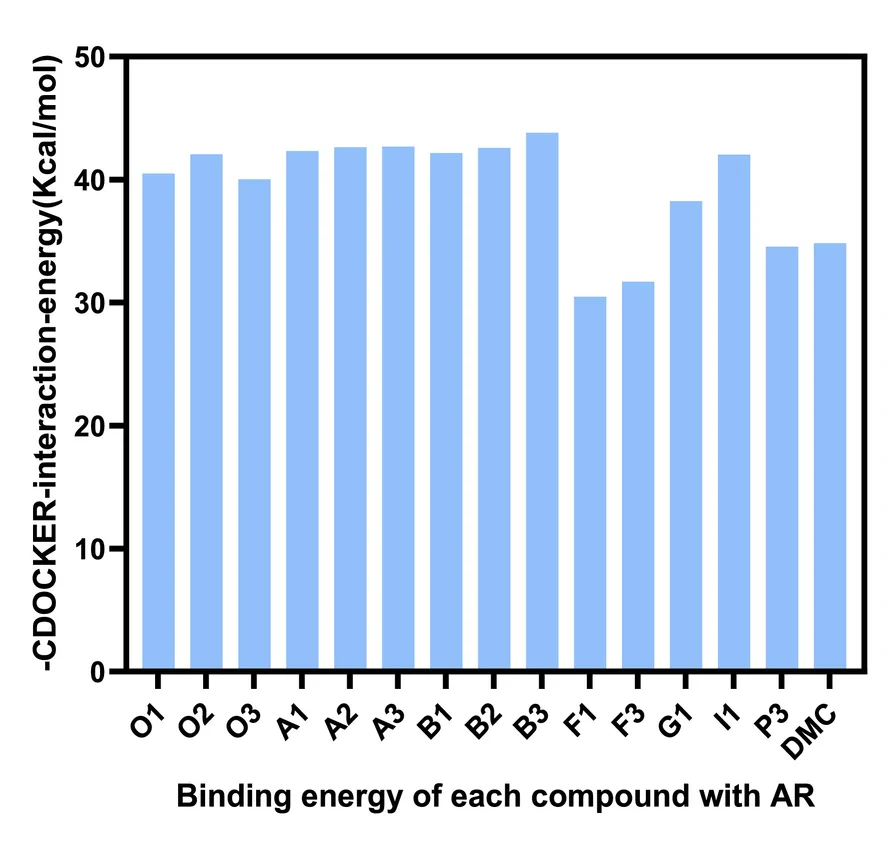
CDOCKER‐Interaction energy of each compound with AR.

The binding modes of compounds A1–A3, B1–B3, O1‐O3, F1, F3, G1, I1, and P3 with AR protein are illustrated in Figures 5, 6, 7, 8, 9, 10, 11, 12, 13. As shown in Figure 5, compound A1 interacts with amino acid residues including TYR834, PHE673, PRO671, LEU830, ASN833, PRO723, and GLU829. Among them, ILE672 forms a π‐alkyl interaction with the benzene ring, TYR834 and PHE673 form π‐alkyl interactions with the cyclopropyl, and PRO671 forms a hydrogen bond with the oxygen atom of acetamido. As shown in Figure 6, compound A2 interacts with amino acid residues such as PHE673, ASN833, ARG840, and GLU837. Among them, GLU837 forms π‐anion and π‐cation interactions with the benzene ring, PHE673 and ARG840 form conventional hydrogen bonds with oxygen atoms of acetamido, and ASN833 forms a conventional hydrogen bond with the hydrogen atom on the nitrogen atom. As shown in Figure 7, compound A3 interacts with amino acid residues including ASN727, ILE672, PRO671, ARG840, PRO723, TYR834, and PHE673. Among them, GLU837 forms π‐anion and π‐cation interactions with the benzene ring, PHE673 and ARG840 form conventional hydrogen bonds with oxygen atoms of acetamido, and ASN833 forms a conventional hydrogen bond with the hydrogen atom attached to the nitrogen atom. As shown in Figure 8, compound I1 interacts with amino acid residues including MET742, MET787, LEU873, MET780, LEU701, and LEU704. Among them, MET742, LEU873, LEU701, LEU704, and VAL746 form π‐alkyl interactions with the benzene ring, MET745, MET749, and LEU707 form alkyl interactions with the cyclopropyl, and MET787 and MET780 form π‐sulfur interactions with the benzene ring. As shown in Figure 9, compound O1 interacts with amino acid residues including ARG840, GLU837, ILE672, LEU830, ASN833, and PHE673. Among them, ARG840 forms conventional hydrogen bonds with the two methoxy groups attached to the benzene ring, GLU837 forms a π‐sulfur interaction with the benzene ring, ILE672 forms a π‐alkyl interaction with the benzene ring, and LEU830 forms an alkyl interaction with the cyclopropyl. As shown in Figure 10, compound O2 interacts with amino acid residues such as GLN670, ARG840, PRO723, ILE672, PHE673, and ASN833. Among them, ARG840 forms a π‐cation interaction with the benzene ring, ILE672 and PHE673 form conventional hydrogen bonds with the methoxy groups on the benzene ring, PRO723 forms an alkyl interaction with the methyl group of the n‐butyl moiety, and ASN833 exhibits a repulsive interaction with the carbon atom of the β‐diketone moiety. As shown in Figure 11, compound O3 interacts with amino acid residues including GLN829, PRO671, TYR834, LEU830, GLU837, PHE673, and ILE672. Among them, GLN829 and PRO671 form van der Waals interactions with the methoxy groups on the benzene ring, ILE672 forms a conventional hydrogen bond with the methoxy group and a π‐alkyl interaction with the benzene ring, TYR834, LEU830, GLU837, and PHE673 form alkyl interactions with the methyl group of the isobutyl moiety. As shown in Figure 12, compound P3 interacts with amino acid residues including ASN727, PHE826, PRO723, GLU837, PHE673, and GLU829. Among them, ASN727, GLU829, and GLU837 form halogen (fluorine) interactions with the trifluoromethyl group on the benzene ring, PHE826 forms an alkyl interaction with the trifluoromethyl group, PRO723 forms an alkyl interaction with the trifluoromethyl group, and GLU829 and GLU837 form π‐anion interactions with the benzene ring. As shown in Figure 13, compound DMC interacts with amino acid residues such as ASN727 and ARG840. Among them, ASN727 forms van der Waals interactions with the methoxy group on the benzene ring, ARG840 forms a conventional hydrogen bond with the oxygen atom and an attractive charge interaction with the carbon atom in the β‐diketone moiety. These results indicated that compounds A1–A3, O1–O3, I1, and P3 displayed different binding modes to AR as compared to DMC.

**FIGURE 5:**
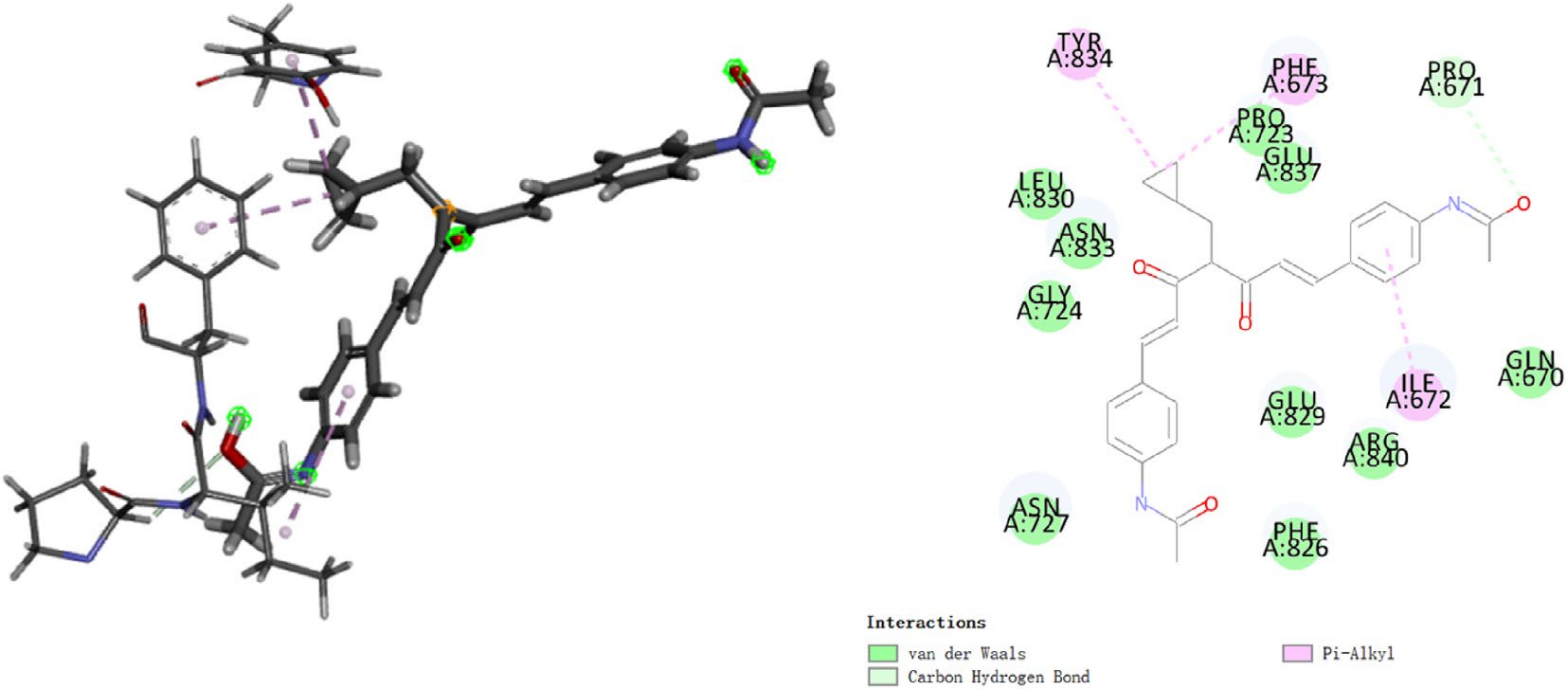
Interaction between A1 and AR (P1: 3D; P2: 2D).

**FIGURE 6:**
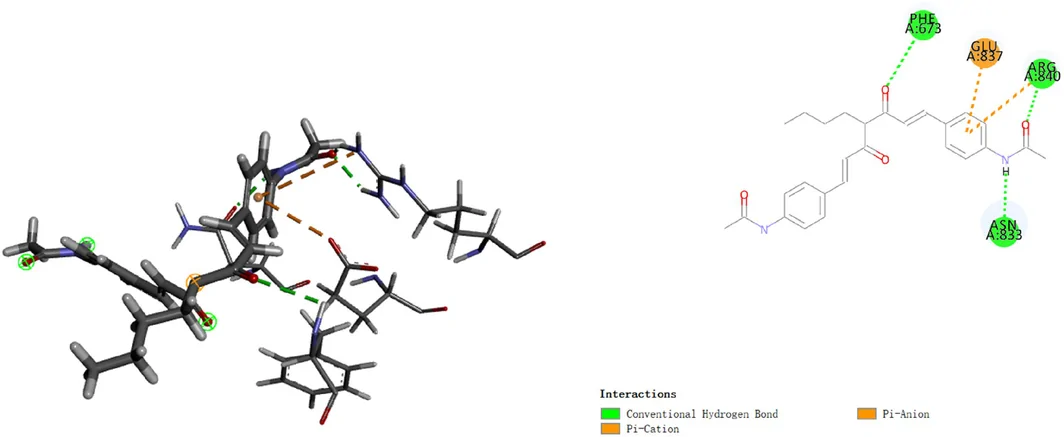
Interaction between A2 and AR (P1: 3D; P2: 2D).

**FIGURE 7:**
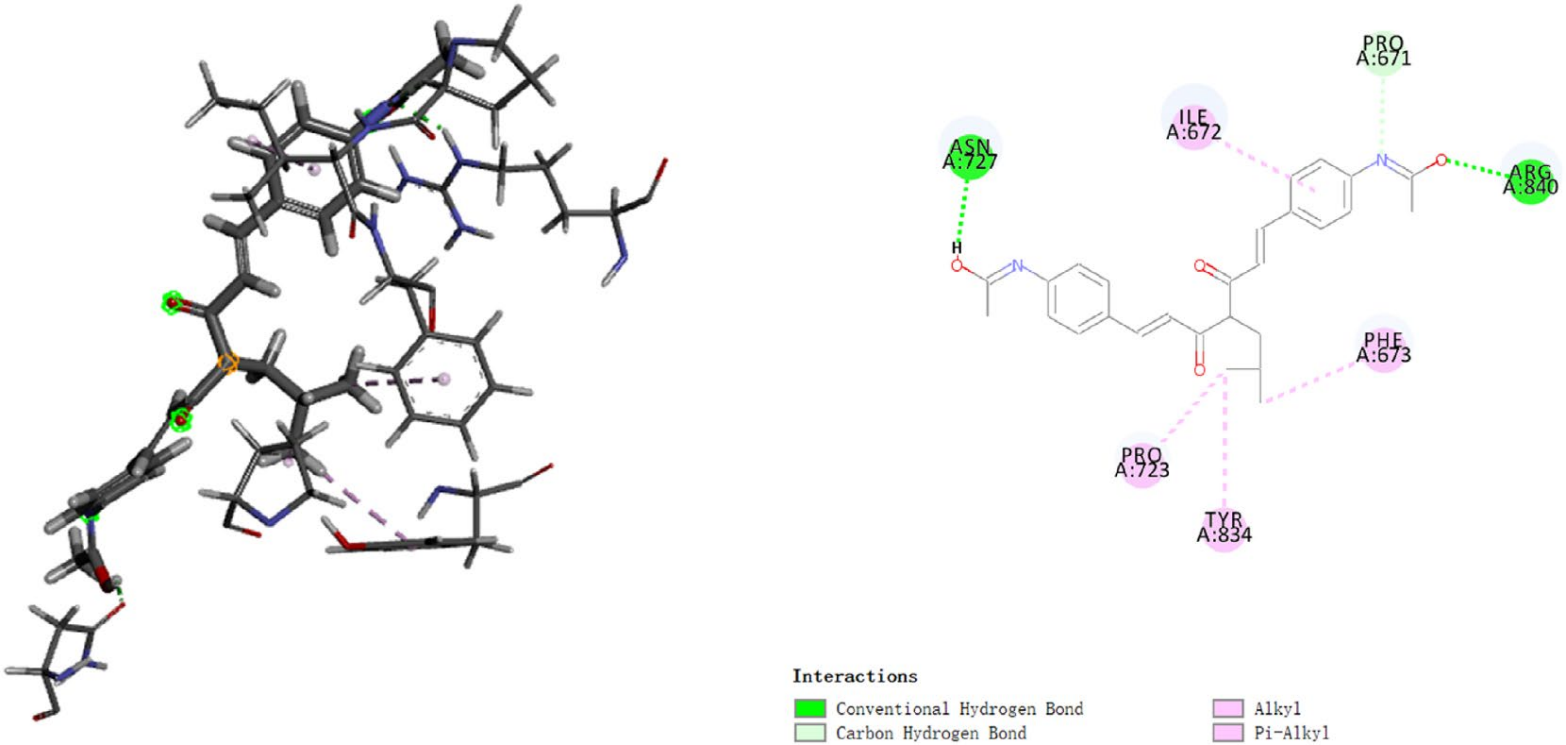
Interaction between A3 and AR (P1: 3D; P2: 2D).

**FIGURE 8:**
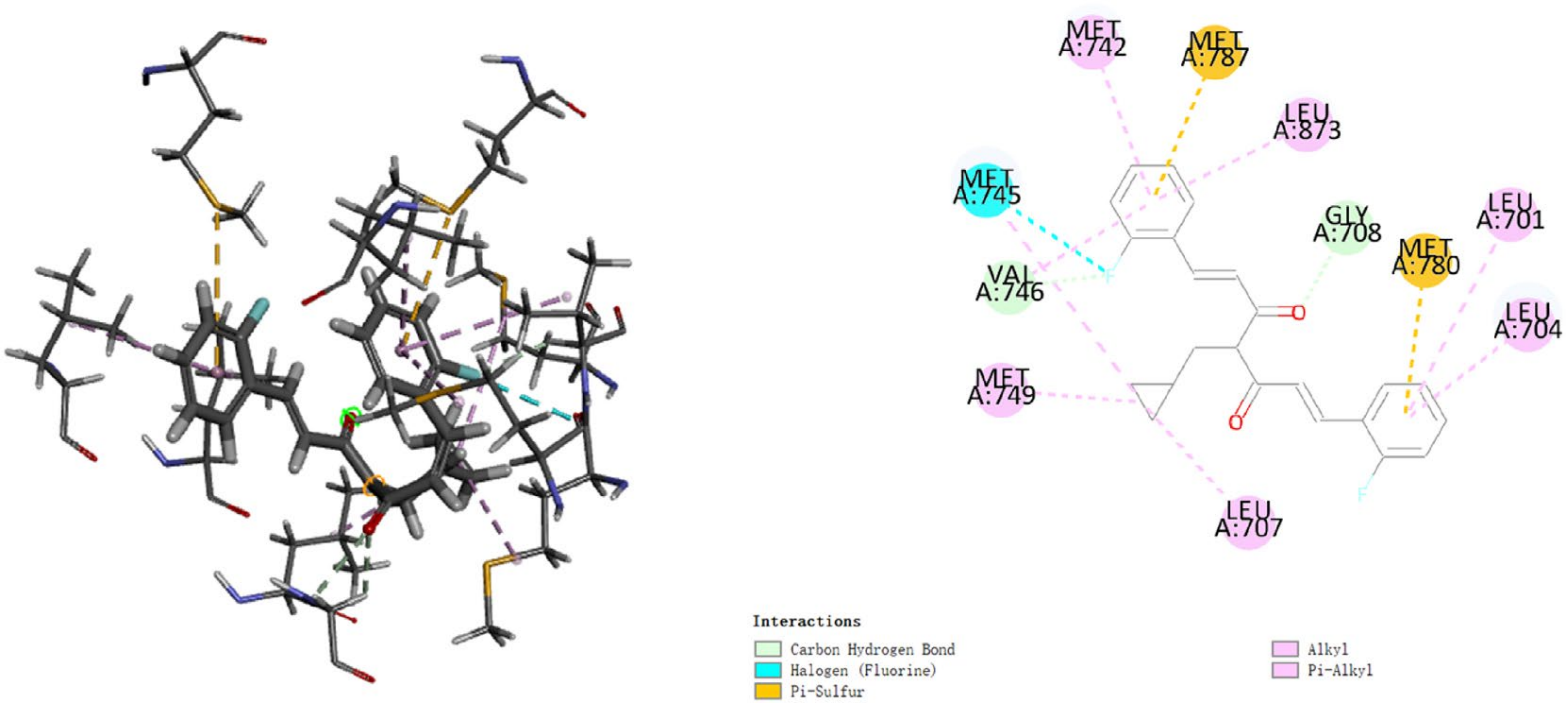
Interaction between I1 and AR (P1: 3D; P2: 2D).

**FIGURE 9:**
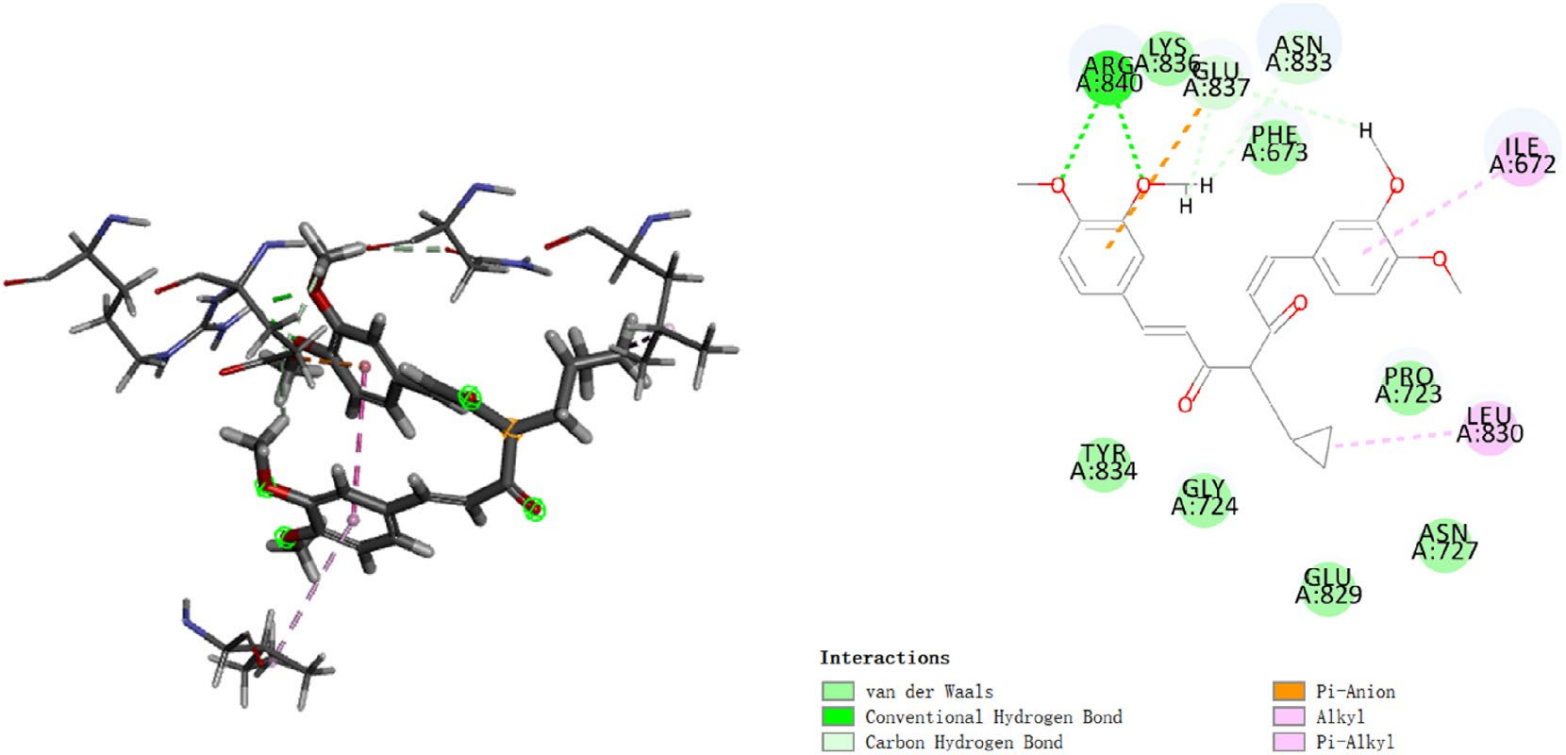
Interaction between O1 and AR (P1: 3D; P2: 2D).

**FIGURE 10:**
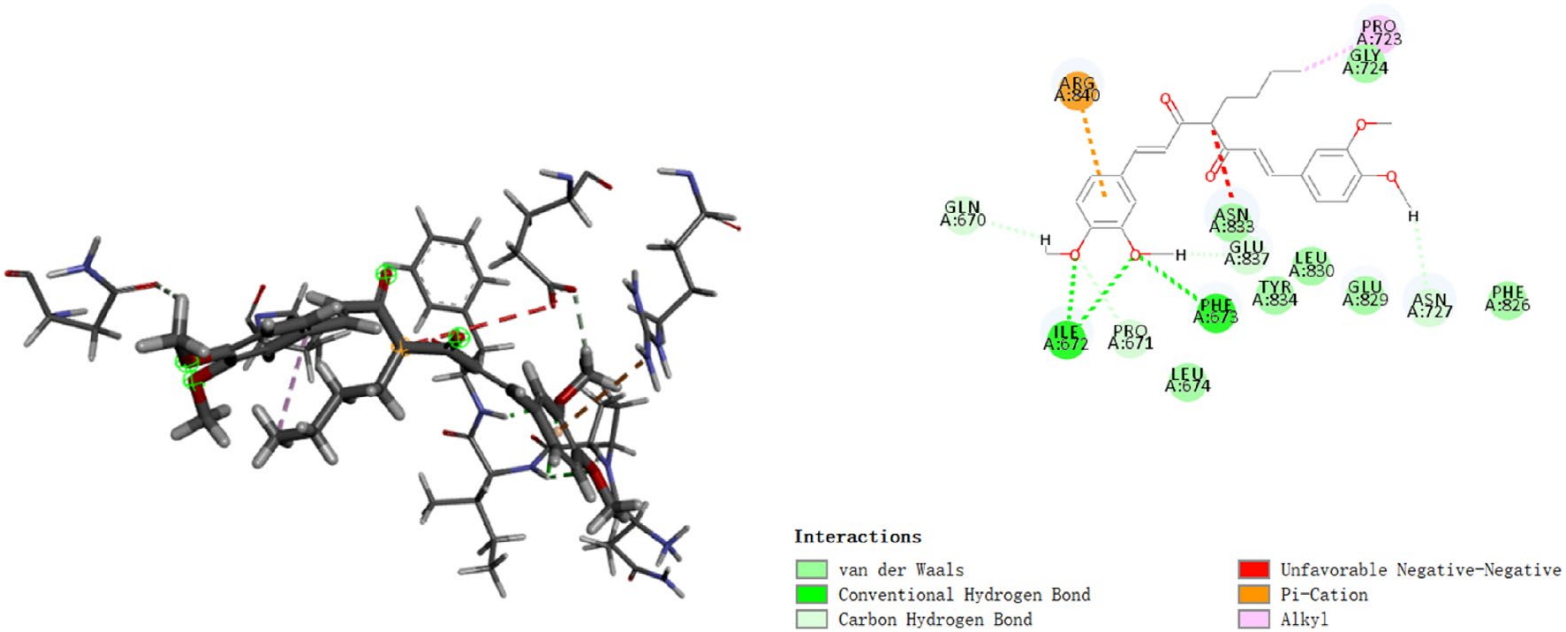
Interaction between O2 and AR (P1: 3D; P2: 2D).

**FIGURE 11:**
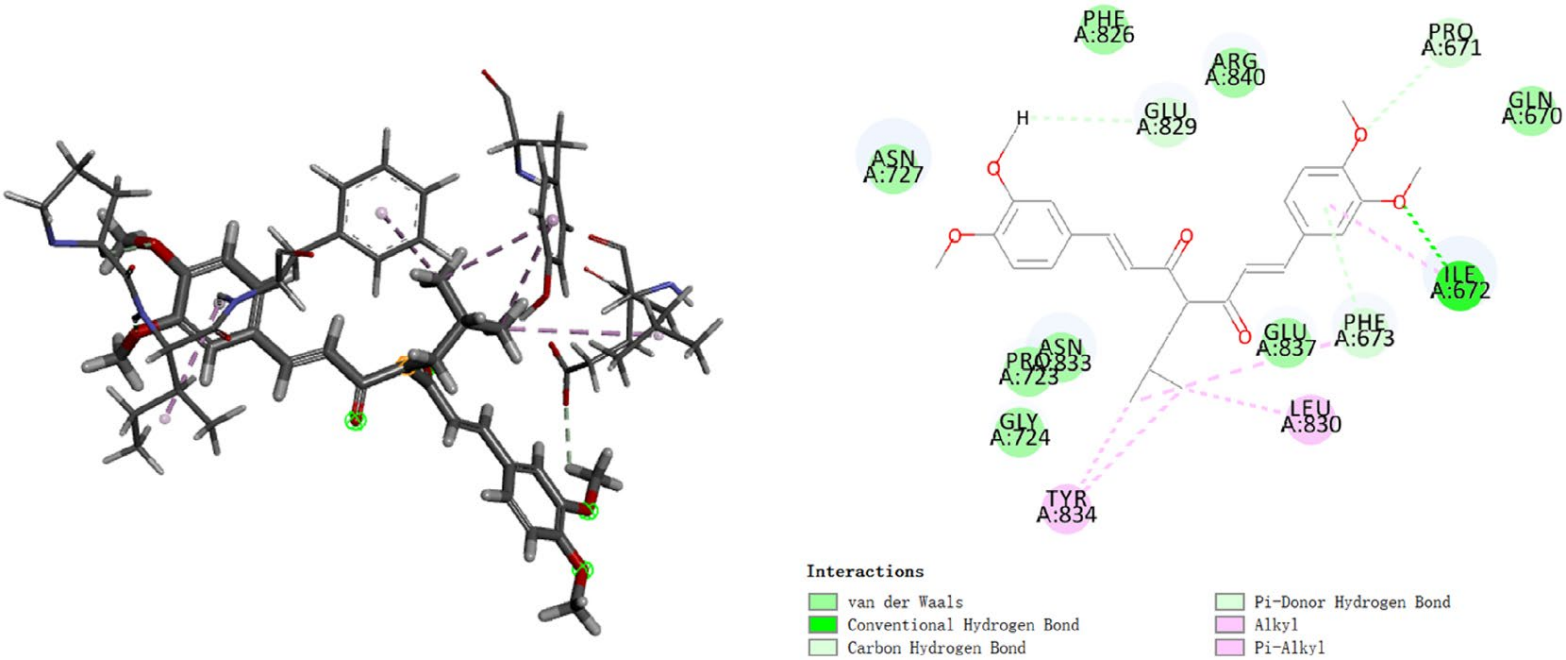
Interaction between O3 and AR (P1: 3D; P2: 2D).

**FIGURE 12:**
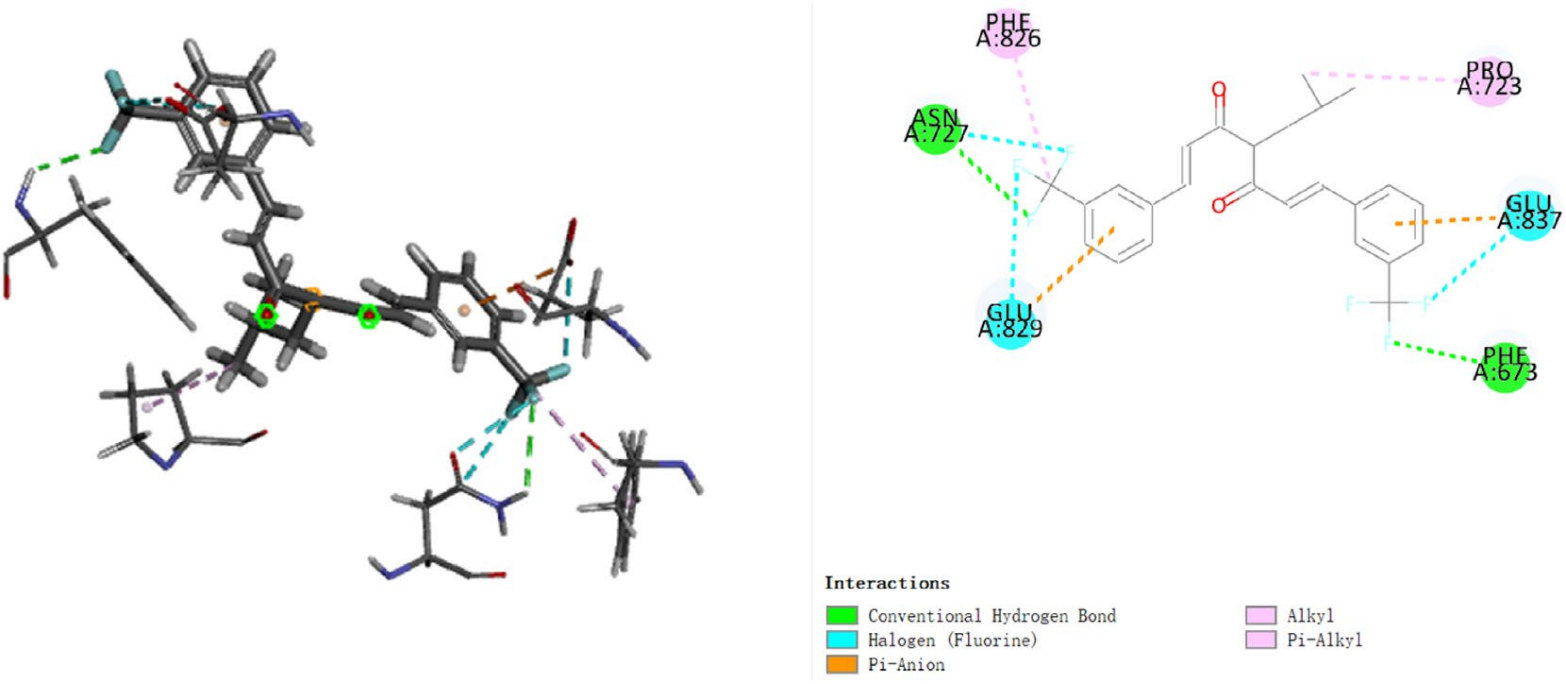
Interaction between P3 and AR (P1: 3D; P2: 2D).

**FIGURE 13:**
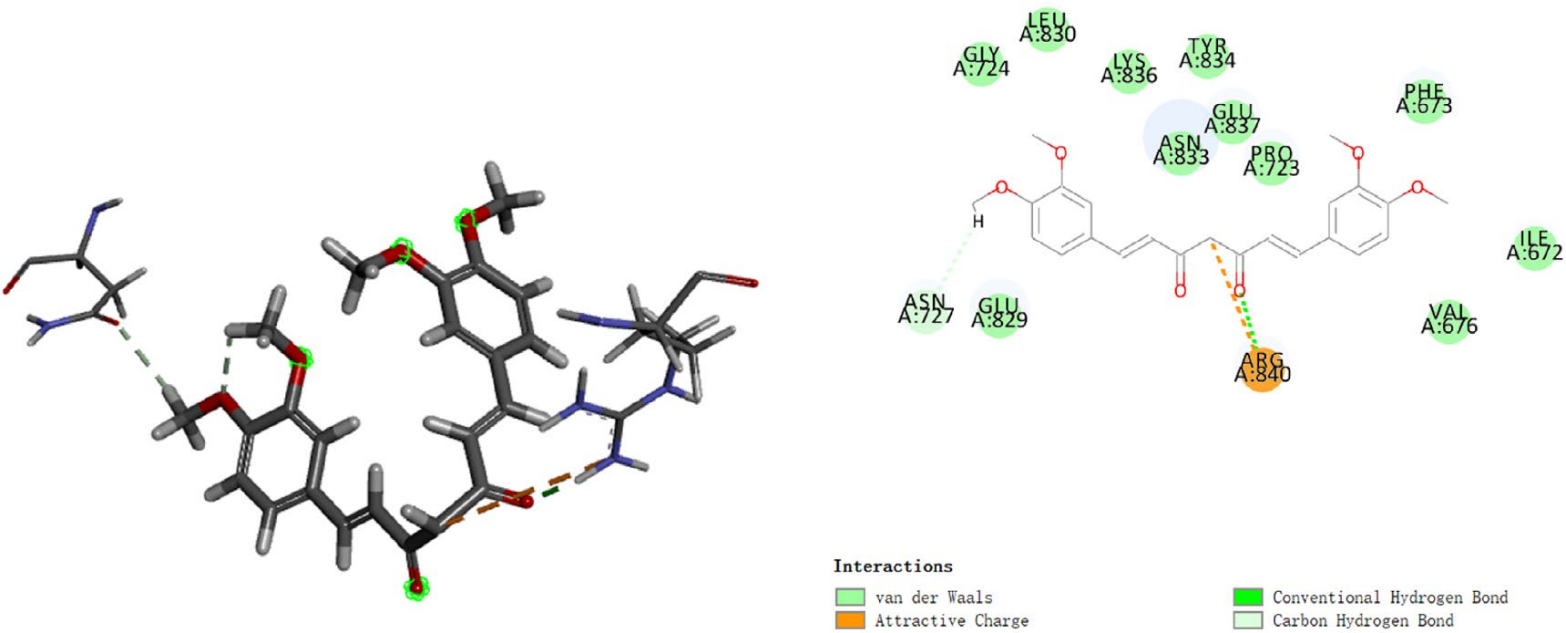
Interaction between DMC and AR (P1: 3D; P2: 2D).

## 4 Conclusion

In this study, we aimed to design and synthesize novel androgen receptor inhibitors against prostate cancer by modifying the chemical structure of curcumin. Among the synthesized compounds, multiple compounds exhibited similar or enhanced cytotoxicity against 22Rv1 cells as compared to DMC. Further cytotoxicity assay on SZ95 cells revealed that compounds A2, A3, B1–B3, F1, F3, G1, and P3 possessed better selectivity toward prostate cancer cells than DMC. The cytotoxicity assay on AR‐negative PC‐3 cells showed that compounds O1–O3, A1–A3, B1–B3, F1, F3, G1, I1, and P3 all exhibited lower cytotoxicity. The structure–activity relationship analysis indicated that both the substituents R_1_ on the β‐diketone moiety and R_2_ on the benzene ring have a significant influence on the activity of the synthesized compounds. The influence of R_2_ on the activity is more prominent. Compounds with 4‐acetamido, 4‐methanesulfonyl, 3‐fluoro, 3,4‐difluoro substituents on the benzene ring are more cytotoxic than compounds with other R_2_ substituents. Western Blot analysis demonstrated that compounds O1–O3, A1–A3, and I1 exerted similar inhibitory effects on both AR‐FL and AR‐V7 of 22Rv1 cells as DMC, while P3 exhibited a significantly enhanced inhibitory effect on AR‐FL. Finally, molecular docking simulations were conducted to investigate the binding affinity and binding modes to AR, which further confirmed that compounds O1–O3, A1–A3, I1, and P3 displayed high targeting ability to AR. Among all synthesized compounds, A2, A3, and P3 stand out with exceptional performance. They not only exhibit high binding affinity to AR, but also potently suppress both full‐length AR and the splice variant AR‐V7. Moreover, these compounds demonstrate selective inhibitory activity against the proliferation of castration‐resistant prostate cancer cells, highlighting their great potential as promising lead candidates for the development of novel castration‐resistant prostate cancer therapeutics.

## Author Contributions

**Manman Huang:** conceptualization, investigation, methodology, writing – original draft, software. **Ziyi Chen:** investigation, validation, software, methodology. **Ruiqi Zhang:** investigation, formal analysis, methodology, data curation. **Shengping Liu:** investigation, methodology. **Defeng Xu:** writing – review and editing, supervision, resources. **Bing Li:** supervision, writing – review and editing, resources, methodology, data curation. **Hang Hu:** writing – review and editing, visualization, supervision, resources, project administration, conceptualization, funding acquisition.

## Funding

This work was supported by Jiangsu Provincial Engineering Research Center for Drug Intelligent Manufacturing and Precision Delivery, MDP1028002.

## Conflicts of Interest

The authors declare no conflicts of interest.

## Supporting information


**Data S1:** Supporting Information.

## Data Availability

Data sharing not applicable to this article as no datasets were generated or analyzed during the current study.
