# Supplementary material for: Synthesis of Curcumin Analogs Targeting Androgen Receptor for the Selective Inhibition of Castration‐Resistant Prostate Cancer Cells
Source: Chem Biol Drug Des. 2026 Oct 15;108(4):e70418. doi: 10.1111/cbdd.70418 (PMC13673116; doi:10.1111/cbdd.70418)
Supplement: Supplementary file 1 [file CBDD-108-e70418-s001.docx]

This file contains the characterization data (part 1), NMR spectra (part 2) and HRMS spectra (part 3) of compounds A1-P3.

1. **Chemical structure, NMR data, HRMS data and purity of compounds A1-P3**

A1

1H NMR (400 MHz, DMSO) δ 10.18 (s, 2H), 7.73 – 7.60 (m, 10H), 7.24 (d, J = 15.4 Hz, 1H), 6.95 (dd, J = 16.0, 1.8 Hz, 1H), 4.66 (t, J = 7.0 Hz, 1H), 2.74 – 2.68 (m, 1H), 2.06 (d, J = 4.0 Hz, 6H), 1.76 (t, J = 7.0 Hz, 1H), 0.93 – 0.63 (m, 1H), 0.38 (ddd, J = 14.1, 9.8, 3.6 Hz, 2H), 0.22 (d, J = 5.1 Hz, 1H), 0.09 (d, J = 4.8 Hz, 1H).

^13^C NMR (101 MHz, DMSO) δ 195.91, 183.60, 183.53, 169.58, 169.15, 169.09, 143.53, 142.18, 141.75, 141.68, 130.15, 130.10, 129.76, 129.68, 124.32, 119.41, 119.35, 111.56, 62.47, 40.57, 40.36, 40.15, 39.95, 39.74, 39.53, 39.32, 33.70, 24.68, 24.58, 13.57, 9.70, 5.26, 4.53.

HRMS: m/z of [M+H]^+^ calculated for C_27_H_28_N_2_O_4_ 445.2127, found 445.2124.

Purity: 98%.

A2

1H NMR (400 MHz, DMSO) δ 10.20 (s, 2H), 7.93 – 7.84 (m, 1H), 7.76 – 7.59 (m, 9H), 7.25 (dd, J = 31.6, 15.4 Hz, 1H), 6.94 (d, J = 16.0 Hz, 1H), 2.75 (d, J = 7.6 Hz, 1H), 2.08 (t, J = 5.0 Hz, 6H), 1.85 (q, J = 7.3 Hz, 1H), 1.49 – 1.19 (m, 4H), 1.00 – 0.82 (m, 3H).

^13^C NMR (101 MHz, DMSO) δ 195.90, 183.08, 177.78, 169.29, 169.09, 143.57, 141.68, 141.26, 130.11, 129.82, 129.68, 124.21, 119.40, 119.34, 112.36, 60.83, 40.57, 40.36, 40.16, 39.95, 39.74, 39.53, 39.32, 35.14, 35.01, 24.77, 24.65, 22.01, 19.10, 14.44, 14.40, 14.31.

HRMS: m/z of [M+H]^+^ calculated for C_27_H_32_N_2_O_4_ 447.2284, found 447.2289.

Purity: 95%.

A3

1H NMR (400 MHz, DMSO) δ 10.20 (d, J = 2.4 Hz, 2H), 7.73 (d, J = 8.4 Hz, 2H), 7.70 – 7.64 (m, 7H), 7.62 (d, J = 6.1 Hz, 1H), 7.22 (d, J = 15.4 Hz, 1H), 6.97 (d, J = 16.0 Hz, 1H), 2.58 (d, J = 7.1 Hz, 1H), 2.08 (d, J = 4.3 Hz, 6H), 1.77 (t, J = 6.9 Hz, 1H), 1.57 (ddt, J = 43.1, 13.3, 6.7 Hz, 1H), 0.94 (dd, J = 22.3, 6.5 Hz, 6H).

^13^C NMR (101 MHz, DMSO) δ 195.92, 183.57, 169.55, 169.48, 143.63, 142.03, 141.45, 141.01, 130.22, 130.10, 129.75, 129.19, 124.13, 119.86, 119.57, 119.48, 111.44, 60.69, 40.49, 40.21, 40.00, 39.79, 39.58, 39.37, 39.17, 38.96, 37.44, 33.31, 31.39, 26.53, 24.45, 22.89, 22.11.

HRMS: m/z of [M+H]^+^ calculated for C_27_H_32_N_2_O_4_ 447.2284, found 447.2284.

Purity: 99%.

B1

^1^H NMR (400 MHz, DMSO) δ 8.14 – 7.92 (m, 8H), 7.78 (dd, *J* = 15.9, 2.5 Hz, 2H), 7.57 (d, *J* = 15.5 Hz, 1H), 7.27 (d, *J* = 16.1 Hz, 1H), 4.78 (t, *J* = 6.9 Hz, 1H), 3.26 (d, *J* = 5.7 Hz, 6H), 2.81 (d, *J* = 6.3 Hz, 1H), 1.82 (t, *J* = 7.0 Hz, 1H), 0.90 – 0.67 (m, 1H), 0.40 (ddd, *J* = 8.3, 5.6, 3.6 Hz, 2H), 0.26 – 0.08 (m, 2H).

^13^C NMR (101 MHz, DMSO) δ 196.28, 183.47, 142.40, 141.87, 141.55, 140.27, 139.83, 139.56, 129.80, 129.69, 129.06, 127.99, 127.96, 125.02, 113.44, 62.67, 49.06, 43.86, 43.77, 40.56, 40.35, 40.14, 39.94, 39.73, 39.52, 39.31, 33.45, 27.63, 13.74, 9.68, 5.35, 4.54.

HRMS: m/z of [M+H]^+^ calculated for C_25_H_26_O_6_S_2_ 487.1249, found 487.1250.

Purity: 96%.

B2

1H NMR (400 MHz, DMSO) δ 8.13 – 7.90 (m, 8H), 7.77 (dd, J = 15.8, 3.2 Hz, 2H), 7.52 (d, J = 15.6 Hz, 2H), 3.26 (s, 6H), 2.76 (s, 2H), 1.41 (h, J = 3.6 Hz, 4H), 0.95 – 0.81 (m, 3H).

^13^C NMR (101 MHz, DMSO) δ 196.25, 183.06, 142.42, 141.88, 141.60, 140.26, 139.84, 139.53, 129.81, 129.65, 129.48, 128.96, 128.00, 127.95, 124.68, 114.13, 43.85, 43.82, 43.77, 40.58, 40.37, 40.16, 39.96, 39.75, 39.54, 39.33, 35.09, 24.54, 22.63, 21.85, 14.45, 14.25, 0.56.

HRMS: m/z of [M+H]^+^ calculated for C_25_H_28_O_6_S_2_ 489.1406, found 489.1407.

Purity: 94%.

B3

1H NMR (400 MHz, DMSO) δ 8.12 – 7.92 (m, 9H), 7.77 (dd, J = 15.8, 3.0 Hz, 2H), 7.53 (d, J = 15.5 Hz, 2H), 4.73 (t, J = 6.9 Hz, 1H), 3.26 (s, 6H), 2.78 – 2.65 (m, 2H), 1.59 (dt, J = 13.5, 6.8 Hz, 1H), 0.93 (dd, J = 11.3, 6.6 Hz, 6H).

^13^C NMR (101 MHz, DMSO) δ 196.00, 183.52, 142.43, 141.85, 141.65, 140.27, 139.61, 139.50, 129.79, 129.62, 128.82, 128.00, 127.96, 125.04, 113.14, 60.83, 49.05, 43.84, 43.76, 40.53, 40.32, 40.12, 39.91, 39.70, 39.49, 39.28, 35.13, 32.96, 31.51, 22.86, 22.01, 14.28.

HRMS: m/z of [M+H]^+^ calculated for C_25_H_28_O_6_S_2_ 489.1406, found 489.1406.

Purity: 95%.

C1

^1^H NMR (600 MHz, DMSO) δ 7.68 (dd, *J* = 15.8, 4.2 Hz, 2H), 7.58 (ddd, *J* = 10.7, 8.4, 2.0 Hz, 2H), 7.42 (ddd, *J* = 8.7, 4.4, 2.0 Hz, 1H), 7.37 – 7.24 (m, 4H), 7.10 (d, *J* = 16.1 Hz, 1H), 4.70 (t, *J* = 6.9 Hz, 1H), 3.90 (d, *J* = 22.4 Hz, 6H), 2.78 (d, *J* = 6.3 Hz, 1H), 1.80 (t, *J* = 7.0 Hz, 1H), 0.89 – 0.65 (m, 1H), 0.39 (ddt, *J* = 10.0, 5.9, 3.0 Hz, 2H), 0.24 – 0.19 (m, 1H), 0.14 – 0.08 (m, 1H).

^13^C NMR (101 MHz, DMSO) δ 195.95, 183.66, 154.68, 154.37, 152.20, 151.89, 148.03, 147.98, 147.92, 147.87, 143.07, 140.94, 132.65, 132.61, 131.77, 126.21, 126.19, 122.94, 122.86, 122.18, 122.11, 121.76, 116.71, 114.30, 113.73, 113.70, 112.19, 62.48, 56.70, 56.58, 40.59, 40.38, 40.17, 39.96, 39.76, 39.55, 39.34, 33.50, 27.59, 13.65, 9.72, 5.26, 4.54.

HRMS: m/z of [M+H]^+^ calculated for C_25_H_24_F_2_O_4_ 427.1721, found 427.1721.

Purity: 97%.

C2

^1^H NMR (400 MHz, DMSO) δ 7.67 (d, *J* = 15.8 Hz, 2H), 7.57 (t, *J* = 6.8 Hz, 2H), 7.44 – 7.24 (m, 5H), 7.09 (d, *J* = 16.1 Hz, 1H), 4.58 (t, *J* = 6.8 Hz, 1H), 3.90 (dd, *J* = 13.4, 1.5 Hz, 6H), 2.73 (s, 1H), 1.87 (q, *J* = 7.3 Hz, 1H), 1.49 – 1.18 (m, 4H), 0.89 (dt, *J* = 28.7, 7.0 Hz, 3H).

^13^C NMR (101 MHz, DMSO) δ 195.99, 183.18, 154.69, 152.20, 151.88, 148.03, 147.96, 147.92, 147.86, 143.09, 140.88, 132.61, 132.57, 126.12, 126.09, 122.11, 116.88, 116.69, 114.07, 114.05, 112.91, 62.01, 56.60, 56.57, 40.58, 40.37, 40.16, 39.95, 39.74, 39.53, 39.33, 34.95, 29.83, 28.24, 24.41, 22.66, 21.82, 14.32, 14.26.

HRMS: m/z of [M+H]^+^ calculated for C_25_H_26_F_2_O_4_ 429.1877, found 429.1882.

Purity: 99%.

C3

^1^H NMR (600 MHz, DMSO) δ 7.67 (dd, *J* = 15.8, 12.9 Hz, 2H), 7.55 (dd, *J* = 8.5, 2.0 Hz, 2H), 7.40 (ddd, *J* = 8.3, 4.4, 2.0 Hz, 1H), 7.35 – 7.24 (m, 4H), 7.10 (d, *J* = 16.0 Hz, 1H), 4.64 (t, *J* = 6.9 Hz, 1H), 3.89 (d, *J* = 20.0 Hz, 6H), 2.63 (d, *J* = 7.0 Hz, 1H), 1.79 (t, *J* = 7.0 Hz, 1H), 1.55 (ddq, *J* = 42.9, 13.5, 6.7 Hz, 1H), 0.92 (dd, *J* = 21.3, 6.6 Hz, 6H).

^13^C NMR (101 MHz, DMSO) δ 195.73, 183.67, 154.70, 154.34, 152.22, 151.87, 148.03, 147.96, 147.85, 143.13, 140.65, 132.64, 132.60, 131.76, 131.73, 125.99, 122.01, 121.95, 121.82, 121.80, 116.89, 116.71, 114.26, 114.23, 113.75, 113.73, 111.97, 60.68, 56.63, 56.57, 40.59, 40.38, 40.17, 39.96, 39.75, 39.54, 39.34, 37.23, 33.05, 31.56, 26.59, 22.94, 22.12.

HRMS: m/z of [M+H]^+^ calculated for C_25_H_26_F_2_O_4_ 429.1877, found 429.1882.

Purity: 96%.

D1

1H NMR (400 MHz, CDCl3) δ 7.72 (dd, J = 15.7, 12.1 Hz, 2H), 7.21 (s, 4H), 7.08 – 6.95 (m, 2H), 2.59 (d, J = 5.8 Hz, 1H), 1.94 (t, J = 7.0 Hz, 1H), 0.95 – 0.66 (m, 1H), 0.50 (p, J = 6.5 Hz, 2H), 0.11 (t, J = 5.4 Hz, 2H).

^13^C NMR (101 MHz, CDCl_3_) δ 194.90, 182.98, 135.12, 132.89, 124.41, 123.52, 112.70, 112.57, 111.36, 77.34, 77.02, 76.70, 33.79, 28.72, 12.34, 9.24, 7.66, 5.00, 4.07.

HRMS: m/z of [M+H]^+^ calculated for C_23_H_16_F_6_O_2_ 439.1133, found 439.1133.

Purity: 95%.

D2

1H NMR (400 MHz, CDCl3) δ 7.63 (dd, J = 15.9, 8.7 Hz, 2H), 7.19 (s, 4H), 7.02 – 6.88 (m, 2H), 2.64 – 2.24 (m, 2H), 1.50 – 1.21 (m, 4H), 0.88 (dt, J = 36.5, 7.2 Hz, 3H).

^13^C NMR (101 MHz, CDCl_3_) δ 194.90, 182.45, 132.92, 124.06, 123.98, 123.62, 123.58, 123.54, 121.41, 112.89, 112.73, 112.56, 77.34, 77.23, 77.02, 76.71, 65.67, 34.48, 29.66, 28.17, 25.63, 22.40, 13.88, 13.81.

HRMS: m/z of [M+H]^+^ calculated for C_23_H_18_F_6_O_2_ 441.1289, found 441.1289.

Purity: 93%.

D3

1H NMR (400 MHz, CDCl3) δ 7.63 (d, J = 15.7 Hz, 2H), 7.19 (s, 2H), 7.16 (s, 1H), 7.12 (s, 1H), 6.95 (tdd, J = 9.1, 6.9, 2.1 Hz, 2H), 2.34 (d, J = 7.1 Hz, 2H), 1.66 (dq, J = 13.4, 6.7 Hz, 1H), 0.93 (d, J = 6.6 Hz, 6H).

^13^C NMR (101 MHz, CDCl_3_) δ 183.10, 153.15, 151.90, 150.62, 149.34, 141.81, 139.30, 139.15, 132.95, 132.92, 132.89, 124.67, 124.59, 123.83, 123.79, 123.75, 123.71, 121.54, 112.88, 112.84, 112.70, 112.66, 112.10, 77.48, 77.16, 76.85, 34.72, 31.41, 22.32, 1.16.

HRMS: m/z of [M+H]^+^ calculated for C_23_H_18_F_6_O_2_ 441.1289, found 441.1284.

Purity: 93%.

F1

^1^H NMR (400 MHz, DMSO) δ 7.80 – 7.55 (m, 6H), 7.52 – 7.40 (m, 3H), 7.26 (qd, *J* = 8.4, 2.6 Hz, 2H), 7.17 (d, *J* = 16.1 Hz, 1H), 4.69 (t, *J* = 6.9 Hz, 1H), 2.77 (d, *J* = 6.4 Hz, 1H), 1.80 (t, *J* = 7.0 Hz, 1H), 0.77 (dtt, *J* = 53.5, 7.4, 5.2 Hz, 1H), 0.43 – 0.32 (m, 2H), 0.25 – 0.18 (m, 1H), 0.15 – 0.07 (m, 1H).

^13^C NMR (101 MHz, DMSO) δ 196.14, 183.56, 164.21, 164.11, 161.79, 161.68, 140.39, 140.36, 138.05, 137.98, 131.43, 131.32, 125.70, 125.67, 125.61, 125.58, 117.42, 117.21, 115.05, 115.00, 114.83, 112.84, 112.82, 40.59, 40.38, 40.17, 39.96, 39.75, 39.55, 39.34, 27.57, 13.75, 4.52.

HRMS: m/z of [M+H]^+^ calculated for C_23_H_20_F_2_O_2_ 367.1510, found 367.1511.

Purity: 98%.

F2

^1^H NMR (400 MHz, DMSO) δ 7.67 (ddd, *J* = 40.3, 20.5, 12.1 Hz, 6H), 7.54 – 7.35 (m, 3H), 7.33 – 7.11 (m, 3H), 4.58 (t, *J* = 6.9 Hz, 1H), 2.74 (d, *J* = 6.6 Hz, 1H), 1.88 (q, *J* = 7.4 Hz, 1H), 1.35 (d, *J* = 52.7 Hz, 4H), 0.89 (dt, *J* = 23.7, 6.8 Hz, 3H).

^13^C NMR (101 MHz, DMSO) δ 196.12, 183.15, 164.20, 164.10, 161.78, 161.68, 142.30, 142.27, 140.40, 138.03, 137.95, 137.25, 131.45, 131.27, 125.67, 125.54, 125.51, 122.85, 115.23, 115.01, 114.84, 113.50, 62.27, 40.59, 40.38, 40.17, 39.96, 39.76, 39.55, 39.34, 35.10, 29.80, 28.15, 24.47, 22.65, 21.86, 14.43, 14.24.

HRMS: m/z of [M+H]^+^ calculated for C_23_H_22_F_2_O_2_ 369.1666, found 369.1667.

Purity: 99%.

F3

1H NMR (400 MHz, DMSO) δ 7.80 – 7.56 (m, 6H), 7.54 – 7.38 (m, 4H), 7.34 – 7.15 (m, 2H), 2.65 (d, J = 7.2 Hz, 2H), 1.61 – 1.44 (m, 1H), 0.94 (dd, J = 13.6, 6.6 Hz, 6H).

^13^C NMR (101 MHz, DMSO) δ 195.92, 183.65, 164.21, 164.10, 161.79, 161.68, 142.33, 140.20, 140.17, 137.97, 131.38, 131.36, 131.27, 127.29, 125.70, 125.53, 123.24, 117.43, 115.24, 115.02, 112.53, 60.90, 40.59, 40.38, 40.17, 39.96, 39.76, 39.55, 39.34, 37.12, 32.85, 31.48, 26.58, 22.89, 22.01. HRMS: m/z of [M+H]^+^ calculated for C_23_H_22_F_2_O_2_ 369.1666, found 369.1667.

Purity: 96%.

G1

^1^H NMR (400 MHz, DMSO) δ 7.83 (ddd, *J* = 12.2, 7.9, 2.1 Hz, 1H), 7.73 (ddd, *J* = 12.0, 7.9, 2.1 Hz, 1H), 7.48 – 7.35 (m, 4H), 7.29 (dtd, *J* = 10.1, 8.4, 1.4 Hz, 2H), 7.17 (d, *J* = 15.5 Hz, 1H), 6.91 (d, *J* = 16.1 Hz, 1H), 4.42 (t, *J* = 6.9 Hz, 1H), 2.54 (d, *J* = 6.4 Hz, 1H), 1.57 (t, *J* = 7.0 Hz, 1H), 0.69 – 0.42 (m, 1H), 0.16 (ddd, *J* = 8.2, 3.5, 2.1 Hz, 2H), -0.06 (dtd, *J* = 40.3, 5.7, 4.0 Hz, 2H).

^13^C NMR (101 MHz, DMSO) δ 183.48, 183.42, 152.16, 152.03, 151.53, 151.40, 149.67, 149.54, 149.09, 148.96, 139.53, 133.44, 133.41, 133.38, 133.34, 126.84, 126.81, 126.77, 126.75, 122.98, 122.95, 118.48, 118.45, 118.31, 117.34, 117.16, 112.78, 40.58, 40.37, 40.16, 39.95, 39.74, 39.54, 39.33, 27.50, 13.81, 5.28, 4.53.

HRMS: m/z of [M+H]^+^ calculated for C_23_H_18_F_4_O_2_ 403.1321, found 403.1317.

Purity: 95%.

G2

^1^H NMR (400 MHz, DMSO) δ 8.03 (ddd, *J* = 12.1, 7.9, 2.1 Hz, 1H), 7.94 (ddd, *J* = 12.0, 7.9, 2.1 Hz, 1H), 7.69 – 7.58 (m, 4H), 7.51 (dt, *J* = 10.5, 8.5 Hz, 2H), 7.35 (d, *J* = 15.5 Hz, 1H), 7.11 (d, *J* = 16.1 Hz, 1H), 4.51 (t, *J* = 6.9 Hz, 1H), 2.75 – 2.67 (m, 1H), 1.86 (q, *J* = 7.2 Hz, 1H), 1.43 – 1.18 (m, 4H), 0.88 (dt, *J* = 20.7, 6.6 Hz, 3H).

^13^C NMR (101 MHz, DMSO) δ 183.03, 152.14, 152.01, 151.51, 151.38, 149.65, 149.53, 149.06, 148.94, 139.49, 133.40, 133.37, 133.34, 133.30, 127.13, 126.70, 126.67, 126.63, 126.60, 122.58, 118.46, 118.29, 117.30, 117.13, 113.36, 113.35, 40.58, 40.37, 40.16, 39.95, 39.74, 39.53, 39.32, 35.11, 24.45, 21.84, 14.43, 14.21, 0.45.

HRMS: m/z of [M+H]^+^ calculated for C_23_H_20_F_4_O_2_ 405.1478, found 405.1473.

Purity: 97%.

G3

1H NMR (400 MHz, DMSO) δ 8.00 (dddd, J = 36.6, 12.1, 7.9, 2.1 Hz, 2H), 7.70 – 7.59 (m, 4H), 7.52 (dtd, J = 10.8, 8.4, 2.8 Hz, 2H), 7.25 (dd, J = 89.1, 15.8 Hz, 2H), 2.64 (d, J = 7.2 Hz, 2H), 1.55 (dp, J = 20.7, 6.7 Hz, 1H), 0.93 (dd, J = 12.3, 6.6 Hz, 6H).

^13^C NMR (101 MHz, DMSO) δ 195.78, 183.57, 151.54, 151.41, 141.49, 139.34, 133.44, 133.37, 133.34, 127.04, 126.77, 126.74, 126.70, 126.67, 123.02, 123.00, 118.64, 118.46, 118.33, 117.55, 117.38, 117.33, 117.15, 112.42, 60.99, 40.59, 40.38, 40.17, 39.96, 39.75, 39.54, 39.34, 37.07, 32.74, 31.43, 26.57, 22.86, 21.97.

HRMS: m/z of [M+H]^+^ calculated for C_23_H_20_F_4_O_2_ 405.1478, found 405.1474.

Purity: 98%.

H1

1H NMR (400 MHz, DMSO) δ 7.91 (t, J = 8.8 Hz, 1H), 7.80 (td, J = 8.9, 2.7 Hz, 1H), 7.71 (d, J = 15.8 Hz, 1H), 7.67 – 7.59 (m, 1H), 7.28 (d, J = 15.6 Hz, 1H), 6.91 – 6.82 (m, 4H), 3.83 (d, J = 1.7 Hz, 6H), 2.67 (d, J = 6.1 Hz, 2H), 0.94 – 0.77 (m, 1H), 0.45 – 0.33 (m, 2H), 0.26 – 0.05 (m, 2H).

^13^C NMR (101 MHz, DMSO) δ 183.45, 183.42, 163.55, 162.93, 162.86, 162.81, 161.05, 161.01, 133.34, 133.31, 130.81, 130.76, 130.50, 121.17, 121.12, 115.66, 115.54, 115.18, 112.07, 112.04, 111.71, 102.40, 102.34, 56.42, 40.58, 40.38, 40.17, 39.96, 39.75, 39.54, 39.33, 27.95, 13.38, 4.49.

HRMS: m/z of [M+H]^+^ calculated for C_25_H_24_F_2_O_4_ 427.1721, found 427.1722.

Purity: 94%.

H2

^1^H NMR (400 MHz, DMSO) δ 7.86 (t, *J* = 8.8 Hz, 2H), 7.69 (d, *J* = 15.6 Hz, 2H), 7.22 (d, *J* = 15.7 Hz, 2H), 6.91 (dd, *J* = 26.6, 10.9 Hz, 4H), 3.82 (s, 6H), 2.59 (s, 2H), 1.40 (s, 4H), 0.93 (s, 3H).

^13^C NMR (101 MHz, DMSO) δ 182.94, 163.58, 162.89, 162.77, 161.08, 133.50, 133.47, 130.94, 130.89, 120.81, 120.75, 115.65, 115.54, 112.50, 112.02, 112.00, 102.38, 102.13, 56.40, 40.59, 40.38, 40.17, 39.96, 39.75, 39.54, 39.33, 34.79, 24.80, 22.08, 14.26.

HRMS: m/z of [M+H]^+^ calculated for C_25_H_26_F_2_O_4_ 429.1877, found 429.1885.

Purity: 95%.

H3

^1^H NMR (400 MHz, DMSO) δ 7.88 (t, *J* = 8.8 Hz, 2H), 7.70 (d, *J* = 15.5 Hz, 2H), 7.25 (d, *J* = 15.6 Hz, 2H), 6.99 – 6.85 (m, 4H), 3.84 (s, 6H), 2.53 (s, 1H), 1.61 (dq, *J* = 13.4, 6.7 Hz, 1H), 0.95 (d, *J* = 6.6 Hz, 7H).

^13^C NMR (101 MHz, DMSO) δ 183.48, 163.57, 162.90, 162.78, 161.07, 133.27, 133.24, 130.94, 130.89, 121.30, 121.24, 115.66, 115.55, 112.07, 112.05, 111.55, 102.40, 102.14, 56.43, 40.58, 40.37, 40.16, 39.96, 39.75, 39.54, 39.33, 33.41, 31.37, 22.16.

HRMS: m/z of [M+H]^+^ calculated for C_25_H_26_F_2_O_4_ 429.1877, found 429.1886.

Purity: 98%.

I1

^1^H NMR (400 MHz, DMSO) δ 7.98 (t, *J* = 7.8 Hz, 1H), 7.88 (t, *J* = 7.7 Hz, 1H), 7.76 (t, *J* = 16.5 Hz, 2H), 7.55 – 7.40 (m, 3H), 7.29 (td, *J* = 11.3, 6.2 Hz, 4H), 7.14 (d, *J* = 16.2 Hz, 1H), 4.76 (t, *J* = 6.9 Hz, 1H), 2.71 (d, *J* = 6.2 Hz, 1H), 1.79 (t, *J* = 7.0 Hz, 1H), 0.92 – 0.65 (m, 1H), 0.41 (dt, *J* = 12.8, 6.5 Hz, 2H), 0.22 (t, *J* = 4.9 Hz, 1H), 0.11 (t, *J* = 4.9 Hz, 1H).

^13^C NMR (101 MHz, DMSO) δ 196.24, 183.43, 162.60, 162.40, 160.09, 159.91, 135.43, 135.40, 133.38, 133.35, 133.26, 132.75, 132.66, 129.90, 129.87, 129.84, 128.30, 128.25, 125.55, 125.51, 125.48, 123.97, 123.92, 123.12, 116.75, 116.67, 116.53, 116.46, 112.59, 112.57, 62.67, 40.59, 40.39, 40.18, 39.97, 39.76, 39.55, 39.34, 33.51, 27.90, 13.43, 9.61, 5.31, 4.50.

HRMS: m/z of [M+H]^+^ calculated for C_23_H_20_F_2_O_2_ 367.1510, found 367.1511.

Purity: 98%.

I2

^1^H NMR (400 MHz, DMSO) δ 8.01 – 7.93 (m, 1H), 7.92 – 7.85 (m, 1H), 7.76 (t, *J* = 15.9 Hz, 2H), 7.50 (td, *J* = 7.7, 3.6 Hz, 2H), 7.41 (d, *J* = 15.7 Hz, 1H), 7.32 (dd, *J* = 13.8, 7.1 Hz, 4H), 7.14 (d, *J* = 16.2 Hz, 1H), 4.64 (t, *J* = 6.9 Hz, 1H), 2.65 (d, *J* = 7.5 Hz, 1H), 1.87 (q, *J* = 7.2 Hz, 1H), 1.50 – 1.20 (m, 4H), 0.90 (dt, *J* = 29.6, 7.0 Hz, 3H).

^13^C NMR (101 MHz, DMSO) δ 196.16, 182.97, 162.43, 159.93, 133.53, 133.49, 133.37, 133.28, 132.71, 132.62, 129.99, 129.96, 125.51, 125.47, 123.63, 123.57, 123.10, 122.99, 122.20, 116.75, 116.69, 116.54, 116.47, 113.34, 40.59, 40.39, 40.18, 39.97, 39.76, 39.55, 39.34, 34.84, 24.76, 22.62, 22.03, 14.27, 14.21.

HRMS: m/z of [M+H]^+^ calculated for C_23_H_22_F_2_O_2_ 369.1666, found 369.1667.

Purity: 97%.

I3

1H NMR (400 MHz, DMSO) δ 7.92 (dt, J = 38.0, 7.7 Hz, 2H), 7.75 (t, J = 16.1 Hz, 2H), 7.55 – 7.25 (m, 8H), 2.57 (d, J = 7.1 Hz, 2H), 1.57 (ddt, J = 38.8, 13.2, 6.7 Hz, 1H), 0.92 (dd, J = 16.3, 6.5 Hz, 6H).

^13^C NMR (101 MHz, DMSO) δ 196.02, 183.49, 162.43, 159.93, 135.52, 133.31, 133.27, 132.72, 132.64, 129.99, 129.97, 125.54, 125.51, 124.08, 124.03, 123.12, 123.01, 116.70, 116.48, 112.38, 61.07, 40.59, 40.39, 40.18, 39.97, 39.76, 39.55, 39.34, 37.16, 33.32, 31.41, 26.59, 22.89, 22.11.

HRMS: m/z of [M+H]^+^ calculated for C_23_H_22_F_2_O_2_ 369.1666, found 369.1671.

Purity: 95%.

J1

^1^H NMR (400 MHz, DMSO) δ 7.85 (ddd, *J* = 23.2, 8.5, 5.6 Hz, 4H), 7.70 (dd, *J* = 15.8, 4.3 Hz, 2H), 7.37 – 7.22 (m, 5H), 7.05 (d, *J* = 16.1 Hz, 1H), 4.70 (t, *J* = 6.9 Hz, 1H), 2.74 (d, *J* = 6.3 Hz, 1H), 1.78 (t, *J* = 7.0 Hz, 1H), 0.76 (dq, *J* = 61.6, 6.4 Hz, 1H), 0.44 – 0.33 (m, 2H), 0.21 (d, *J* = 5.0 Hz, 1H), 0.10 (d, *J* = 4.9 Hz, 1H).

^13^C NMR (101 MHz, DMSO) δ 196.05, 183.62, 164.88, 162.41, 140.44, 132.16, 132.13, 131.57, 131.48, 131.36, 131.28, 121.59, 121.57, 116.63, 116.53, 116.41, 116.31, 112.10, 40.59, 40.39, 40.18, 39.97, 39.76, 39.55, 39.34, 27.69, 13.61, 9.68, 5.28, 4.50.

HRMS: m/z of [M+H]^+^ calculated for C_23_H_20_F_2_O_2_ 367.1510, found 367.1509.

Purity: 99%.

J2

^1^H NMR (400 MHz, DMSO) δ 7.85 (dtd, *J* = 20.6, 5.7, 2.2 Hz, 4H), 7.74 – 7.62 (m, 2H), 7.33 – 7.26 (m, 5H), 6.99 (dd, *J* = 40.4, 16.0 Hz, 1H), 4.58 (t, *J* = 6.9 Hz, 1H), 2.69 (s, 1H), 1.86 (q, *J* = 7.2 Hz, 1H), 1.48 – 1.18 (m, 4H), 0.89 (dt, *J* = 26.5, 7.1 Hz, 3H).

^13^C NMR (101 MHz, DMSO) δ 196.03, 183.63, 183.18, 164.87, 162.43, 162.40, 142.58, 140.46, 139.63, 132.14, 132.11, 131.82, 131.79, 131.57, 131.48, 131.32, 131.28, 131.23, 131.16, 131.08, 124.61, 121.20, 121.17, 116.64, 116.59, 116.54, 116.42, 116.38, 116.32, 112.84, 102.29, 40.59, 40.39, 40.18, 39.97, 39.76, 39.55, 39.34, 35.02, 24.61, 22.64, 21.94, 14.43, 14.24.

HRMS: m/z of [M+H]^+^ calculated for C_23_H_22_F_2_O_2_ 369.1666, found 369.1668.

Purity: 98%.

J3

^1^H NMR (400 MHz, DMSO) δ 7.86 (ddd, *J* = 19.0, 8.4, 5.6 Hz, 4H), 7.71 (dd, *J* = 15.8, 8.3 Hz, 2H), 7.34 – 7.25 (m, 5H), 7.08 (d, *J* = 16.1 Hz, 1H), 4.66 (t, *J* = 6.9 Hz, 1H), 2.62 (d, *J* = 7.1 Hz, 1H), 1.79 (t, *J* = 6.9 Hz, 1H), 1.57 (ddt, *J* = 33.4, 13.4, 6.7 Hz, 1H), 0.94 (dd, *J* = 16.1, 6.5 Hz, 6H).

^13^C NMR (101 MHz, DMSO) δ 183.67, 164.86, 162.39, 140.23, 132.15, 132.12, 131.30, 131.21, 121.60, 121.58, 116.52, 116.31, 111.84, 40.60, 40.39, 40.18, 39.97, 39.76, 39.55, 39.34, 33.09, 31.42, 22.08.

HRMS: m/z of [M+H]^+^ calculated for C_23_H_22_F_2_O_2_ 369.1666, found 369.1668.

Purity: 98%.

M1

^1^H NMR (400 MHz, DMSO) δ 8.09 (q, *J* = 8.2 Hz, 1H), 7.97 (q, *J* = 8.2 Hz, 1H), 7.70 (t, *J* = 16.3 Hz, 2H), 7.45 – 7.33 (m, 3H), 7.21 (q, *J* = 7.5 Hz, 2H), 7.11 (d, *J* = 16.2 Hz, 1H), 4.72 (t, *J* = 6.9 Hz, 1H), 2.70 (d, *J* = 6.3 Hz, 1H), 1.78 (t, *J* = 7.0 Hz, 1H), 0.91 – 0.63 (m, 1H), 0.39 (dd, *J* = 11.5, 7.6 Hz, 2H), 0.20 (d, *J* = 5.0 Hz, 1H), 0.10 (d, *J* = 4.9 Hz, 1H).

^13^C NMR (101 MHz, DMSO) δ 196.13, 183.37, 183.34, 134.53, 132.42, 131.65, 131.50, 127.97, 123.63, 119.12, 113.19, 112.97, 112.58, 105.50, 105.24, 105.12, 104.98, 104.86, 62.69, 40.60, 40.39, 40.19, 39.98, 39.77, 39.56, 39.35, 33.46, 27.82, 13.47, 9.60, 5.30, 4.51.

HRMS: m/z of [M+H]^+^ calculated for C_23_H_18_F_4_O_2_ 403.1321, found 403.1326.

Purity: 93%.

M2

^1^H NMR (400 MHz, DMSO) δ 8.06 (q, *J* = 8.2 Hz, 1H), 7.97 (q, *J* = 8.1 Hz, 1H), 7.69 (t, *J* = 15.0 Hz, 2H), 7.43 – 7.32 (m, 3H), 7.22 (td, *J* = 8.5, 3.1 Hz, 2H), 7.10 (d, *J* = 16.2 Hz, 1H), 2.63 (d, *J* = 7.4 Hz, 1H), 1.86 (q, *J* = 7.2 Hz, 1H), 1.46 – 1.18 (m, 4H), 0.89 (dt, *J* = 27.8, 6.9 Hz, 3H).

^13^C NMR (101 MHz, DMSO) δ 182.92, 162.64, 162.55, 160.24, 160.12, 132.58, 131.65, 131.51, 123.25, 120.00, 119.96, 119.89, 113.31, 113.13, 113.10, 112.91, 105.41, 105.15, 104.89, 40.59, 40.38, 40.17, 39.96, 39.75, 39.54, 39.34, 34.86, 24.68, 22.02, 14.30.

HRMS: m/z of [M+H]^+^ calculated for C_23_H_20_F_4_O_2_ 405.1478, found 405.1485.

Purity: 94%.

M3

^1^H NMR (400 MHz, CDCl_3_) δ 7.68 (d, *J* = 15.7 Hz, 2H), 7.45 (td, *J* = 8.6, 6.6 Hz, 2H), 7.11 (d, *J* = 15.6 Hz, 2H), 6.90 – 6.76 (m, 4H), 2.34 (d, *J* = 7.1 Hz, 2H), 1.67 (hept, *J* = 6.7 Hz, 1H), 0.90 (dd, *J* = 22.0, 6.7 Hz, 6H).

^13^C NMR (101 MHz, CDCl_3_) δ 183.24, 164.80, 163.13, 163.01, 162.28, 160.58, 160.47, 133.35, 130.97, 130.92, 130.87, 130.82, 123.42, 123.34, 120.15, 120.03, 112.19, 112.15, 111.98, 111.94, 111.57, 105.02, 104.76, 104.51, 77.34, 77.03, 76.71, 34.60, 31.23, 22.55, 22.22.

HRMS: m/z of [M+H]^+^ calculated for C_23_H_20_F_4_O_2_ 405.1478, found 405.1479.

Purity: 93%.

N1

^1^H NMR (400 MHz, DMSO) δ 8.19 (d, *J* = 7.9 Hz, 1H), 7.99 – 7.91 (m, 1H), 7.89 – 7.73 (m, 7H), 7.64 (q, *J* = 6.3 Hz, 3H), 4.64 (t, *J* = 6.9 Hz, 1H), 2.78 (d, *J* = 6.3 Hz, 1H), 1.82 (t, *J* = 7.0 Hz, 1H), 0.90 – 0.68 (m, 1H), 0.41 (q, *J* = 6.0 Hz, 2H), 0.16 (dq, *J* = 37.2, 4.9 Hz, 2H).

^13^C NMR (101 MHz, DMSO) δ 195.00, 182.14, 181.97, 136.39, 134.81, 133.86, 132.46, 132.17, 130.12, 129.75, 129.64, 129.01, 128.11, 127.91, 127.83, 127.78, 126.71, 126.41, 125.70, 125.65, 124.87, 112.28, 102.83, 62.24, 39.56, 39.35, 39.14, 38.93, 38.72, 38.51, 38.31, 32.28, 26.51, 12.58, 8.55, 4.23, 3.47.

HRMS: m/z of [M+H]^+^ calculated for C_25_H_20_F_6_O_2_ 467.1446, found 467.1446.

Purity: 95%.

N2

^1^H NMR (400 MHz, DMSO) δ 8.05 (dd, *J* = 94.1, 11.6 Hz, 4H), 7.78 (dt, *J* = 25.8, 8.4 Hz, 5H), 7.64 (q, *J* = 6.7 Hz, 2H), 7.41 (d, *J* = 15.2 Hz, 1H), 2.71 (d, *J* = 7.4 Hz, 2H), 1.34 (d, *J* = 50.6 Hz, 4H), 0.87 (dt, *J* = 19.5, 7.0 Hz, 3H).

^13^C NMR (101 MHz, DMSO) δ 196.01, 182.74, 137.52, 135.88, 133.58, 133.49, 132.81, 131.15, 130.64, 129.94, 129.08, 128.92, 128.73, 128.12, 127.82, 127.75, 127.45, 126.73, 126.68, 126.62, 126.56, 126.00, 125.53, 123.28, 114.03, 62.96, 40.60, 40.39, 40.18, 39.97, 39.77, 39.56, 39.35, 34.99, 29.78, 27.88, 24.54, 22.60, 21.94, 14.35, 14.17.

HRMS: m/z of [M+H]^+^ calculated for C_25_H_22_F_6_O_2_ 469.1602, found 469.1606.

Purity: 94%.

N3

^1^H NMR (400 MHz, DMSO) δ 8.19 (d, *J* = 7.9 Hz, 2H), 8.00 – 7.91 (m, 2H), 7.87 – 7.73 (m, 4H), 7.66 (q, *J* = 7.3 Hz, 2H), 7.45 (d, *J* = 15.3 Hz, 2H), 4.61 (t, *J* = 6.9 Hz, 1H), 2.66 (d, *J* = 7.1 Hz, 2H), 1.60 (tp, *J* = 12.9, 6.8 Hz, 1H), 0.94 (dd, *J* = 13.0, 6.5 Hz, 6H).

^13^C NMR (101 MHz, DMSO) δ 195.82, 183.23, 137.61, 135.67, 133.59, 133.47, 132.79, 131.14, 130.59, 129.81, 129.06, 128.94, 128.71, 128.12, 128.04, 127.83, 127.75, 127.46, 127.16, 126.70, 126.65, 126.59, 126.53, 125.99, 125.87, 123.27, 120.55, 113.00, 61.42, 40.60, 40.39, 40.18, 39.97, 39.76, 39.56, 39.35, 36.93, 32.94, 31.46, 26.60, 22.74, 22.01.

HRMS: m/z of [M+H]^+^ calculated for C_25_H_22_F_6_O_2_ 469.1602, found 469.1611.

Purity: 93%.

O1

^1^H NMR (600 MHz, DMSO) δ 7.65 (dd, *J* = 15.7, 11.1 Hz, 2H), 7.39 – 7.19 (m, 5H), 7.03 – 6.96 (m, 3H), 4.67 (t, *J* = 7.0 Hz, 1H), 3.87 – 3.77 (m, 12H), 1.78 (t, *J* = 7.0 Hz, 2H), 0.94 – 0.59 (m, 1H), 0.39 (dddd, *J* = 12.0, 8.1, 5.8, 4.0 Hz, 2H), 0.26 – 0.04 (m, 2H).

^13^C NMR (101 MHz, DMSO) δ 195.70, 151.80, 151.37, 149.44, 144.19, 141.80, 128.42, 127.42, 124.10, 124.04, 123.25, 119.43, 112.18, 112.05, 111.64, 110.92, 62.43, 56.20, 56.04, 40.58, 40.37, 40.16, 39.95, 39.74, 39.54, 39.33, 33.60, 9.74, 5.22, 4.54.

HRMS: m/z of [M+H]^+^ calculated for C_27_H_30_O_6_ 451.2121, found 451.2128.

Purity: 95%.

O2

^1^H NMR (600 MHz, DMSO) δ 7.68 – 7.56 (m, 2H), 7.36 – 7.31 (m, 4H), 7.27 (dt, *J* = 8.4, 2.6 Hz, 1H), 7.17 (d, *J* = 15.4 Hz, 1H), 7.01 (dd, *J* = 8.3, 2.7 Hz, 2H), 4.55 (t, *J* = 6.9 Hz, 1H), 3.82 (dd, *J* = 12.6, 1.8 Hz, 12H), 2.69 (t, *J* = 7.2 Hz, 1H), 1.85 (q, *J* = 7.2 Hz, 1H), 1.50 – 1.20 (m, 4H), 0.90 (dt, *J* = 54.5, 7.1 Hz, 3H).

^13^C NMR (101 MHz, DMSO) δ 188.44, 187.88, 156.17, 156.11, 154.22, 154.18, 146.48, 145.65, 133.12, 132.76, 128.18, 128.01, 127.24, 123.77, 116.93, 116.91, 116.85, 116.11, 115.60, 106.26, 60.83, 60.80, 45.33, 45.18, 45.12, 44.92, 44.71, 44.50, 44.29, 44.08, 39.65, 29.22, 26.62, 19.06.

HRMS: m/z of [M+H]^+^ calculated for C_27_H_32_O_6_ 453.2277, found 453.2285.

Purity: 92%.

O3

^1^H NMR (600 MHz, DMSO) δ 7.63 (d, *J* = 15.4 Hz, 2H), 7.37 – 7.31 (m, 4H), 7.18 (d, *J* = 15.4 Hz, 2H), 7.02 (d, *J* = 8.3 Hz, 2H), 4.62 (t, *J* = 6.9 Hz, 1H), 3.83 (d, *J* = 14.4 Hz, 12H), 2.61 (d, *J* = 7.0 Hz, 2H), 1.57 (dtt, *J* = 59.5, 13.4, 6.7 Hz, 1H), 0.96 (d, *J* = 6.6 Hz, 6H).

^13^C NMR (101 MHz, DMSO) δ 195.51, 183.63, 151.83, 151.34, 149.43, 144.21, 141.50, 128.40, 127.38, 124.10, 123.85, 123.09, 119.51, 112.21, 112.06, 111.54, 110.93, 60.60, 56.12, 56.05, 56.03, 40.58, 40.37, 40.16, 39.95, 39.74, 39.53, 39.33, 37.39, 31.59, 26.57, 23.02, 22.22.

HRMS: m/z of [M+H]^+^ calculated for C_27_H_32_O_6_ 453.2277, found 453.2287.

Purity: 96%.

P1

^1^H NMR (400 MHz, DMSO) δ 8.06 (s, 1H), 7.99 (d, *J* = 9.4 Hz, 2H), 7.92 (d, *J* = 7.9 Hz, 1H), 7.71 – 7.49 (m, 6H), 7.41 (d, *J* = 15.5 Hz, 1H), 7.16 (d, *J* = 16.2 Hz, 1H), 4.65 (t, *J* = 6.9 Hz, 1H), 2.69 (d, *J* = 6.3 Hz, 1H), 1.69 (t, *J* = 6.9 Hz, 1H), 0.78 – 0.54 (m, 1H), 0.26 (dq, *J* = 8.2, 3.0 Hz, 2H), 0.13 – -0.06 (m, 2H).

^13^C NMR (101 MHz, DMSO) δ 196.10, 183.63, 141.87, 140.12, 136.62, 135.88, 132.83, 132.69, 130.52, 130.46, 130.42, 130.14, 128.02, 127.36, 126.81, 125.88, 125.77, 125.65, 123.79, 113.03, 62.75, 40.60, 40.40, 40.19, 39.98, 39.77, 39.56, 39.35, 33.33, 27.45, 13.75, 9.71, 5.28, 4.52.

HRMS: m/z of [M+H]^+^ calculated for C_25_H_20_F_6_O_2_ 467.1446, found 467.1444.

Purity: 97%.

P2

^1^H NMR (400 MHz, DMSO) δ 8.18 (s, 1H), 8.17 – 8.10 (m, 2H), 8.07 (d, *J* = 7.9 Hz, 1H), 7.84 – 7.74 (m, 4H), 7.68 (q, *J* = 7.2 Hz, 2H), 7.51 (d, *J* = 15.6 Hz, 1H), 7.30 (d, *J* = 16.1 Hz, 1H), 4.65 (t, *J* = 6.8 Hz, 1H), 2.77 (d, *J* = 7.2 Hz, 1H), 1.91 (q, *J* = 7.2 Hz, 1H), 1.42 (p, *J* = 5.7 Hz, 2H), 1.31 (tt, *J* = 17.4, 7.8 Hz, 2H), 0.90 (dt, *J* = 22.3, 7.0 Hz, 3H).

^13^C NMR (101 MHz, DMSO) δ 196.08, 183.16, 141.88, 140.03, 136.59, 135.85, 132.81, 132.58, 130.77, 130.50, 130.46, 130.40, 130.14, 129.82, 128.56, 127.93, 127.34, 126.79, 125.85, 125.76, 125.63, 125.59, 125.56, 125.53, 123.42, 123.14, 123.05, 113.69, 62.32, 40.60, 40.39, 40.18, 39.97, 39.77, 39.56, 39.35, 34.99, 29.82, 28.07, 24.33, 22.63, 21.77, 14.31, 14.23.

HRMS: m/z of [M+H]^+^ calculated for C_25_H_22_F_6_O_2_ 469.1602, found 469.1602.

Purity: 99%.

P3

^1^H NMR (400 MHz, DMSO) δ 8.19 – 8.11 (m, 3H), 8.07 (d, *J* = 7.9 Hz, 1H), 7.80 (dd, *J* = 16.4, 9.5 Hz, 4H), 7.69 (q, *J* = 7.2 Hz, 2H), 7.51 (d, *J* = 15.6 Hz, 1H), 7.32 (d, *J* = 16.1 Hz, 1H), 4.75 (t, *J* = 6.9 Hz, 1H), 2.69 (d, *J* = 7.0 Hz, 1H), 1.82 (t, *J* = 6.9 Hz, 1H), 1.57 (dp, *J* = 20.6, 6.7 Hz, 1H), 0.94 (dd, *J* = 10.5, 6.6 Hz, 6H).

^13^C NMR (101 MHz, DMSO) δ 196.08, 183.16, 141.88, 140.03, 136.59, 135.85, 132.81, 132.58, 130.77, 130.50, 130.46, 130.40, 130.14, 129.82, 128.56, 127.93, 127.34, 126.79, 125.85, 125.76, 125.63, 125.59, 125.56, 125.53, 123.42, 123.14, 123.05, 113.69, 62.32, 40.60, 40.39, 40.18, 39.97, 39.77, 39.56, 39.35, 34.99, 29.82, 28.07, 24.33, 22.63, 21.77, 14.31, 14.23.

HRMS: m/z of [M+H]^+^ calculated for C_25_H_22_F_6_O_2_ 469.1602, found 469.1602.

Purity: 98%.

2. **^1^H NMR and ^13^C NMR spectra of compounds A1-P3**

^1^H NMR spectrum of A1

^13^C NMR spectrum of A1

^1^H NMR spectrum of A2

^13^C NMR spectrum of A2

^1^H NMR spectrum of A3

^13^C NMR spectrum of A3

^1^H NMR spectrum of B1

^13^C NMR spectrum of B1

^1^H NMR spectrum of B2

^13^C NMR spectrum of B2

^1^H NMR spectrum of B3

^13^C NMR spectrum of B3

^1^H NMR spectrum of C1

^13^C NMR spectrum of C1

^1^H NMR spectrum of C2

^13^C NMR spectrum of C2

^1^H NMR spectrum of C3

^13^C NMR spectrum of C3

^1^H NMR spectrum of D1

^13^C NMR spectrum of D1

^1^H NMR spectrum of D2

^13^C NMR spectrum of D2

^1^H NMR spectrum of D3

^13^C NMR spectrum of D3

^1^H NMR spectrum of F1

^13^C NMR spectrum of F1

^1^H NMR spectrum of F2

^13^C NMR spectrum of F2

^1^H NMR spectrum of F3

^13^C NMR spectrum of F3

^1^H NMR spectrum of G1

^13^C NMR spectrum of G1

^1^H NMR spectrum of G2

^13^C NMR spectrum of G2

^1^H NMR spectrum of G3

^13^C NMR spectrum of G3

^1^H NMR spectrum of H1

^13^C NMR spectrum of H1

^1^H NMR spectrum of H2

^13^C NMR spectrum of H2

^1^H NMR spectrum of H3

^13^C NMR spectrum of H3

^1^H NMR spectrum of I1

^13^C NMR spectrum of I1

^1^H NMR spectrum of I2

^13^C NMR spectrum of I2

^1^H NMR spectrum of I3

^13^C NMR spectrum of I3

^1^H NMR spectrum of J1

^13^C NMR spectrum of J1

^1^H NMR spectrum of J2

^13^C NMR spectrum of J2

^1^H NMR spectrum of J3

^13^C NMR spectrum of J3

^1^H NMR spectrum of M1

^13^C NMR spectrum of M1

^1^H NMR spectrum of M2

^13^C NMR spectrum of M2

^1^H NMR spectrum of M3

^13^C NMR spectrum of M3

^1^H NMR spectrum of N1

^13^C NMR spectrum of N1

^1^H NMR spectrum of N2

^13^C NMR spectrum of N2

^1^H NMR spectrum of N3

^13^C NMR spectrum of N3

^1^H NMR spectrum of O1

^13^C NMR spectrum of O1

^1^H NMR spectrum of O2

^13^C NMR spectrum of O2

^1^H NMR spectrum of O3

^13^C NMR spectrum of O3

^1^H NMR spectrum of P1

^13^C NMR spectrum of P1

^1^H NMR spectrum of P2

^13^C NMR spectrum of P2

^1^H NMR spectrum of P3

^13^C NMR spectrum of P3

3. **HRMS spectra of compounds A1-P3**

HRMS spectrum of A1

HRMS spectrum of A2

HRMS spectrum of A3

HRMS spectrum of B1

HRMS spectrum of B2

HRMS spectrum of B3

HRMS spectrum of C1

HRMS spectrum of C2

HRMS spectrum of C3

HRMS spectrum of D1

HRMS spectrum of D2

HRMS spectrum of D3

HRMS spectrum of F1

HRMS spectrum of F2

HRMS spectrum of F3

HRMS spectrum of G1

HRMS spectrum of G2

HRMS spectrum of G3

HRMS spectrum of H1

HRMS spectrum of H2

HRMS spectrum of H3

HRMS spectrum of I1

HRMS spectrum of I2

HRMS spectrum of I3

HRMS spectrum of J1

HRMS spectrum of J2

HRMS spectrum of J3

HRMS spectrum of M1

HRMS spectrum of M2

HRMS spectrum of M3

HRMS spectrum of N1

HRMS spectrum of N2

HRMS spectrum of N3

HRMS spectrum of O1

HRMS spectrum of O2

HRMS spectrum of O3

HRMS spectrum of P1

HRMS spectrum of P2

HRMS spectrum of P3
